# The MDM2–p53–pyruvate carboxylase signalling axis couples mitochondrial metabolism to glucose-stimulated insulin secretion in pancreatic β-cells

**DOI:** 10.1038/ncomms11740

**Published:** 2016-06-06

**Authors:** Xiaomu Li, Kenneth K. Y. Cheng, Zhuohao Liu, Jin-Kui Yang, Baile Wang, Xue Jiang, Yawen Zhou, Philip Hallenborg, Ruby L. C. Hoo, Karen S. L. Lam, Yasuhiro Ikeda, Xin Gao, Aimin Xu

**Affiliations:** 1 State Key Laboratory of Pharmaceutical Biotechnology, The University of Hong Kong, Pok Fu Lam, Hong Kong; 2 Department of Medicine, The University of Hong Kong, Pok Fu Lam, Hong Kong; 3 Department of Endocrinology and Metabolism, Zhongshan Hospital, Fudan University, Shanghai 20032, China; 4 Beijing Key Laboratory of Diabetes Research and Care, Beijing Tongren Hospital, Capital Medical University, Beijing 100730, China; 5 Department of Biochemistry and Molecular Biology, University of Southern Denmark, Odense 5230, Denmark; 6 Department of Molecular Medicine, Mayo Clinic, Rochester, Minnesota 55905, USA; 7 Department of Pharmacology & Pharmacy, The University of Hong Kong, Pok Fu Lam, Hong Kong

## Abstract

Mitochondrial metabolism is pivotal for glucose-stimulated insulin secretion (GSIS) in pancreatic β-cells. However, little is known about the molecular machinery that controls the homeostasis of intermediary metabolites in mitochondria. Here we show that the activation of p53 in β-cells, by genetic deletion or pharmacological inhibition of its negative regulator MDM2, impairs GSIS, leading to glucose intolerance in mice. Mechanistically, p53 activation represses the expression of the mitochondrial enzyme pyruvate carboxylase (PC), resulting in diminished production of the TCA cycle intermediates oxaloacetate and NADPH, and impaired oxygen consumption. The defective GSIS and mitochondrial metabolism in MDM2-null islets can be rescued by restoring PC expression. Under diabetogenic conditions, MDM2 and p53 are upregulated, whereas PC is reduced in mouse β-cells. Pharmacological inhibition of p53 alleviates defective GSIS in diabetic islets by restoring PC expression. Thus, the MDM2–p53–PC signalling axis links mitochondrial metabolism to insulin secretion and glucose homeostasis, and could represent a therapeutic target in diabetes.

## Abstract (web summary)

Mice lacking the tumour suppressor p53 are partially protected from developing diabetes. Here the authors show that p53 is upregulated in the pancreas of diabetic mice where it impairs β cell function by repressing expression of mitochondrial pyruvate carboxylase, thereby inhibiting insulin secretion.

## Graphical abstract

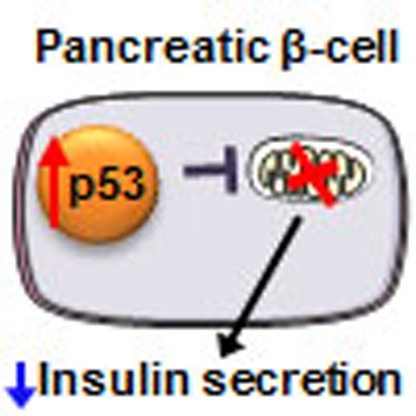

Glucose-stimulated insulin secretion (GSIS) is tightly controlled by a complex metabolic process involving mitochondrial oxidative metabolism in pancreatic β-cells^1^,^2^. Dysregulation of this process contributes to the development of diabetes^3^. Mitochondria act as fuel sensors and generators that couple glucose metabolism to insulin secretion by producing numerous coupling factors through the tricarboxylic acid (TCA) cycle. In mitochondria, pyruvate derived from glycolysis is metabolized to generate NADH and FADH_2_, which are subsequently oxidized via the respiration chain to produce ATP. An increase of ATP/ADP ratio induces closure of the ATP-sensitive potassium (K_ATP_) channel, leading to membrane depolarization and calcium influx, which in turn result in first-phase insulin secretion^1^. On the other hand, the byproducts such as NADPH, α-ketoglutarate and GTP generated during mitochondrial pyruvate cycling in the TCA cycle act as amplifying factors for second-phase insulin secretion^4^. These metabolic pathways are coordinated by various mitochondrial enzymes, such as malic enzymes, pyruvate dehydrogenase kinase and pyruvate carboxylase (PC)^4^, yet their molecular regulation and precise roles in the pathogenesis of β-cell dysfunction in diabetes remain largely unknown.

The transcription factor p53 is a well-established tumour suppressor that can be activated by a wide range of stresses, for example, DNA damage and oxidative stress^5^. Emerging evidence has suggested a role for p53 in obesity- and ageing-related cardiometabolic disorders, such as insulin resistance, endothelial dysfunction, liver and heart diseases^5^,^6^,^7^,^8^,^9^,^10^. In obesity, augmented p53 expression in adipose tissues causes metabolic inflammation by triggering cellular senescence. p53 activity is upregulated in pancreatic β-cells in diabetic patients and rodents, and is induced by diabetogenic agents such as streptozotocin and palmitic acid in primary mouse islets^11^,^12^,^13^. Systemic overexpression of the short isoform of p53 (which stabilizes p53 and mediates its activation) reduces β-cell proliferation, leading to glucose intolerance and hypoinsulinaemia in mice^14^. On the contrary, global knockout of p53 alleviates streptozotocin-induced diabetes, at least in part, by preserving β-cell mass^12^. Despite these promising findings, interpretation of the data from the global p53 transgenic and knockout mice is hindered by the fact that the p53 actions in other metabolic organs may also contribute to the altered metabolic phenotypes. Therefore, the roles of p53 in β-cell function, especially GSIS, are yet to be clearly defined.

The activity of p53 is tightly controlled by its upstream negative regulator mouse double minute 2 (MDM2). This ubiquitin E3 ligase promotes proteasomal degradation and blocks the transcriptional activity of p53 through the direct interaction and ubiquitination^15^. As *MDM2* itself is a downstream target of p53, induction of its expression by p53 results in an autoregulatory negative feedback loop that returns MDM2 and p53 to a basal level. Perturbations in their balance not only contribute to cancer development but also to metabolic disorders^5^. For instance, genetic abrogation of *MDM2* in hepatocytes causes unrestrained p53 activation, leading to liver fibrosis in mice^7^. In addition, disruption of this feedback loop perturbs metabolic adaption to starvation, resulting in fatty liver disease^6^.

Here we employ β-cell-specific genetic knockout and pharmacological blockade approaches to investigate the role of the MDM2–p53 axis in β-cell functions. Our results show that the MDM2–p53 axis is essential for mitochondrial oxidative metabolism and subsequent GSIS in β-cells by regulating the mitochondrial enzyme PC. Furthermore, we explore the feasibility to reverse impaired GSIS in type 2 diabetic mice by pharmacological intervention of the MDM2–p53 signalling axis.

## Results

### Inhibition of MDM2 impairs GSIS and glucose tolerance

**Figure 1:**
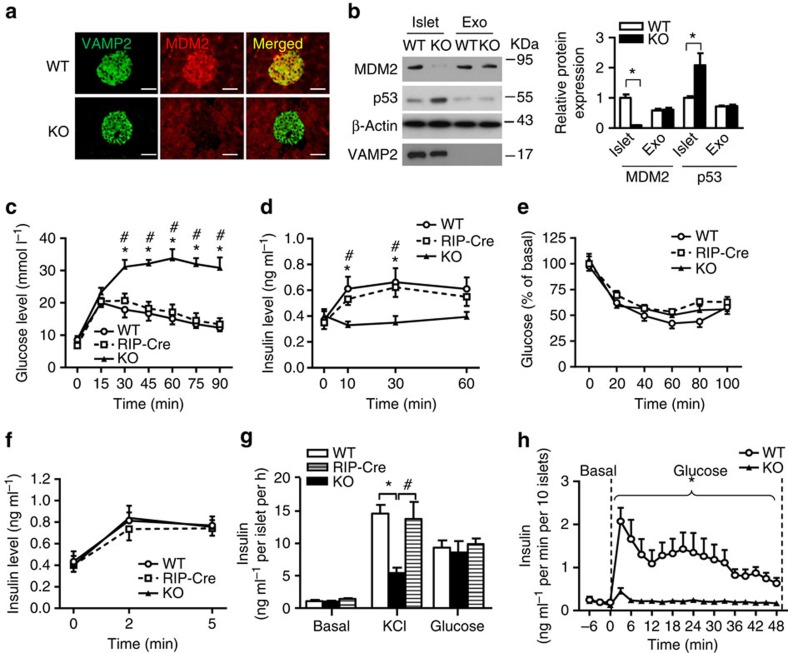
Effects of β-cell-specific deletion of *MDM2* on glucose homeostasis and insulin secretion in mice. (**a**) Immunofluorescence staining of MDM2 (red) and VAMP2 (as a β-cell marker) (green) in pancreatic sections of 6-week-old male β-cell-specific MDM2 knockout (β-MDM2KO) mice and its WT littermates with original magnification at × 400. (**b**) Pancreatic islets and exocrine (Exo) cells isolated from the above mice were subjected to immunoblotting using an antibody against MDM2, p53, β-actin or VAMP2 as indicated. The right panel is the densitometric analysis for the relative abundance of MDM2 and p53 normalized with β-actin. (**c**–**h**) Twelve-week-old male β-MDM2KO mice, WT littermates and RIP-Cre controls were used (*n*=6). (**c**) GTT. (**d**) Serum insulin levels during the GTT in panel C. (**e**) ITT. (**f**) Serum insulin levels on L-arginine stimulation. (**g**) The isolated islets were stimulated with glucose (20 mM) or KCl (20 mM) for 30 min, followed by measurement of insulin concentration in the conditional medium (*n*=5). (**h**) Dynamic insulin secretion of the isolated islets in response to glucose stimulation (10 mM) using a perfusion system (*n*=4). Note that the islets were maintained in the conditional medium with 2.8 mM glucose as basal levels and the insulin content of the isolated islets was comparable between the three genotypes. All experiments were repeated at least four times and representative images are shown. *P<0.05 (KO versus WT), ^#^*P*<0.05 (KO versus Cre control) (Student's *t*-test). Scale bar, 50 μm. All data are represented as the mean±s.e.m.

To determine whether disruption of the MDM2–p53 axis has any impact on β-cell functions, we generated β-cell-specific MDM2 knockout (β-MDM2KO) mice by crossing *MDM2*^*floxed/floxed*^ mice with transgenic mice expressing Cre recombinase under the control of rat insulin II promoter (RIP-Cre mice)^16^. Immunofluorescence staining and immunoblotting revealed a marked decrease of MDM2 protein to a virtually undetectable level in pancreatic islets, but not in exocrine cells of β-MDM2KO mice (Fig. 1a,b). On the contrary, protein level of p53 was significantly upregulated by ∼2.1-fold in islets of β-MDM2KO mice when compared with those in wild-type (WT) littermates (MDM2^floxed^ mice without Cre) (Fig. 1b). In addition, MDM2 null islets displayed a moderate but statistically significant increase in mRNA level of *p21* (the canonical p53 target), compared with WT controls (Supplementary Fig. 1a). On the other hand, immunoblotting revealed that there was no obvious difference in expression of hypothalamic MDM2 and p53 between the two groups of mice (Supplementary Fig. 1b). Genetic deletion of *MDM2* had no obvious effect on food intake, body weight, insulin and glucose levels under fasting conditions (Supplementary Table 1). However, male β-MDM2KO mice exhibited a marked impairment in glucose tolerance on glucose challenge when compared with their WT littermates or RIP-Cre controls (Fig. 1c). The impaired glucose tolerance in male β-MDM2KO mice was associated with defective GSIS and independent of insulin sensitivity, as determined by insulin tolerance test (ITT; Fig. 1d,e). Likewise, glucose intolerance and defective GSIS were also observed in female β-MDM2KO mice (Supplementary Fig. 2). On the other hand, insulin secretion induced by L-arginine was comparable between β-MDM2KO mice and WT controls (Fig. 1f). Consistent with the *in vivo* observations, our *ex vivo* analysis in isolated islets showed that total, first and second phases of GSIS were sharply diminished by β-cell-specific ablation of *MDM2*, whereas insulin secretion induced by potassium chloride (KCl) was similar between MDM2-deficient islets and WT controls (Fig. 1g,h and Supplementary Fig. 3). Since WT littermates and RIP-Cre controls exhibited similar phenotypes in terms of glucose tolerance and insulin secretion, we therefore only included WT littermates for comparison in all subsequent experiments.

**Figure 2:**
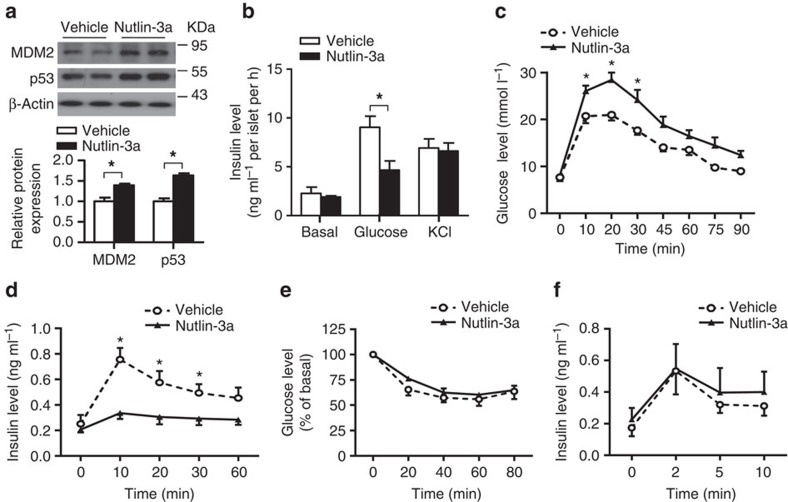
Effects of the MDM2 inhibitor nutlin-3a on glucose metabolism and insulin secretion in mice. (**a**) Islets isolated from 12-week-old male C57 mice were treated with nutlin-3a (10 μg ml^−1^) or DMSO as vehicle for 6 h, followed by immunoblotting using an anti-MDM2, -p53 or -β-actin antibody. Representative immunoblot images from three independent experiments are shown. The lower panel is the densitometric analysis for the relative abundance of MDM2 and p53 normalized with β-actin. (**b**) The islets treated with nutlin-3a or vehicle were subjected to static GSIS assay as in Fig. 1g. Note that the insulin content of islets treated with vehicle or nutlin-3a was similar. (**c**–**f**) Twelve-week-old male C57 mice were treated with nutlin-3a (10 mg kg^−1^) or DMSO as vehicle by oral gavage, followed by fasting for 6 h. (**c**) GTT. (**d**) Serum insulin levels during the GTT in **c**. (**e**) ITT. (**f**) Serum insulin levels during L-arginine stimulation test. *P<0.05 (*n*=6) (Student's *t*-test). All data are represented as the mean±s.e.m.

To test whether pharmacological inhibition of MDM2 impairs GSIS, we treated C57 mice with nutlin-3a, a small molecule MDM2 inhibitor that activates p53 by blocking the MDM2–p53 interaction. Treatment with nutlin-3a led to a marked induction of MDM2 and p53 expression in pancreatic islets, accompanied with reduction of GSIS (Fig. 2a,b). Furthermore, the mice treated with nutlin-3a exhibited severe glucose intolerance and defective GSIS, but normal insulin sensitivity as determined by ITT (Fig. 2c–e). Similar to the β-MDM2KO mice, treatment with nutlin-3a significantly attenuated glucose- but not L-arginine- or KCl-induced insulin secretion in mice or isolated islets, respectively (Fig. 2b,f).

### Inactivation of p53 alleviates MDM2-null phenotypes

**Figure 3:**
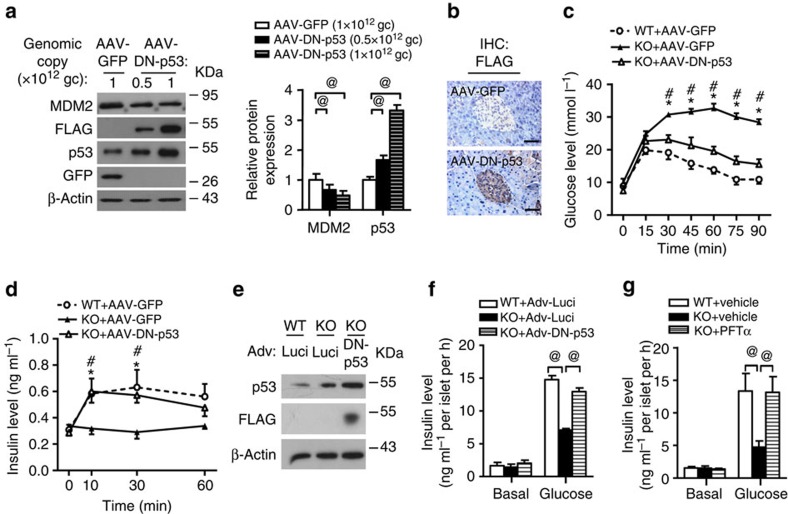
Inhibition of p53 in β-cells improves glucose tolerance and insulin secretion in β-MDM2KO mice. (**a**) Pancreatic islets were isolated from C57 mice intraperitoneally injected with AAV encoding FLAG-tagged dominant-negative form of p53 (AAV-DN-p53) or GFP control (AAV-GFP) at an indicated amount of genomic copy (gc) number per mouse for 2 weeks. The islets were subjected to immunoblotting using an antibody against MDM2, FLAG, p53, GFP or β-actin as indicated. The right panel is the densitometric analysis for the relative abundance of MDM2 and p53 normalized with β-actin (*n*=4). (**b**) IHC staining with an anti-FLAG antibody in pancreatic sections of C57 mice injected with AAV-GFP or AAV-DN-p53 (1 × 10^12^ gc per mouse) with original magnification at × 400. Scale bar, 50 μm. (**c**–**d**) Eight-week-old male β-MDM2KO mice and its WT littermates were injected with AAV-GFP or AAV-DN-p53 at a dose of 1 × 10^12^ gc per mouse for 2 weeks. (**c**) GTT. (**d**) Serum insulin levels during GTT in **c**. (**e**–**g**) Isolated islets from β-MDM2KO mice or its WT littermates were infected with adenovirus encoding luciferase (Adv-Luci) or FLAG-tagged DN-p53 (Adv-DN-p53) (**e**,**f**) or treated with PFTα (10 μM) or DMSO as vehicle control (**g**) for 24 h. (**e**) The infected islets were subjected to immunoblotting with an anti-p53, -FLAG or -β-actin antibody. (**f**,**g**) Static GSIS in the islets were measured at 30 min after glucose stimulation (20 mM) (*n*=5). Note that insulin content in the isolated islets was similar between different treatment groups. *P<0.05 (KO+AAV-GFP versus WT+AAV-GFP, *n*=6), ^#^*P*<0.05 (KO+AAV-GFP versus KO+AAV-DN-p53, *n*=6), ^@^*P*<0.05 (Student's *t*-test). All experiments were repeated at least three times and representative immunoblots and IHC images are shown. All data are represented as the mean±s.e.m.

Since genetic disruption or pharmacological inhibition of MDM2 led to p53 activation in pancreatic β-cells, we next explored whether the inactivation of p53 could reverse glucose intolerance and defective GSIS in β-MDM2KO mice. To this end, we injected β-MDM2KO mice with adeno-associated virus (AAV) expressing FLAG-tagged dominant-negative mutant of p53 (DN-p53) (in which arginine-172 of p53 was mutated to histidine (p53^R172H^)) under the control of a modified mouse insulin promoter^17^,^18^. Of note, the p53^R172H^ mutant has been shown to silence WT p53 activities by blocking the binding ability of p53 to the DNA responsive elements of its target gene^17^,^18^, and the efficiency of our AAV system was previously validated^19^. Immunoblotting analysis revealed that the p53 protein level was increased by approximately threefold relative to its endogenous levels in islets of mice infected with AAV encoding FLAG-tagged DN-p53. This was accompanied with a reduction of MDM2 protein level (Fig. 3a). In addition, expression of FLAG-tagged DN-p53 was confirmed by immunohistochemical staining of pancreatic tissues using an anti-FLAG antibody (Fig. 3b). AAV-mediated inactivation of p53 partially reversed glucose intolerance and defective GSIS in β-MDM2KO mice (Fig. 3c,d). Of note, AAV-mediated inactivation of p53 had no impact on β-cell mass, as determined by haematoxylin and eosin staining (Supplementary Fig. 4). Moreover, inhibition of p53 either by adenovirus-mediated overexpression of DN-p53 or treatment with the p53 inhibitor pifithrin-α (PFTα) reversed defective GSIS in MDM2-deficient islets (Fig. 3e–g).

**Figure 4:**
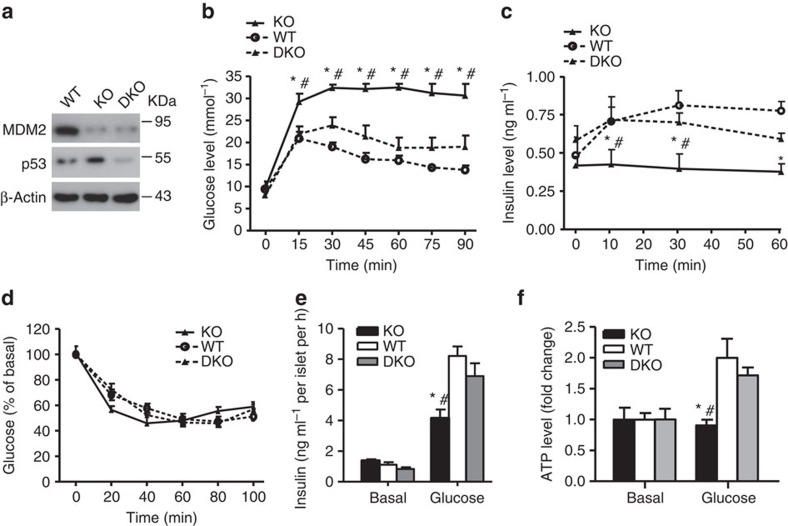
β-cell-specific deletion of *p53* partially reverses glucose intolerance and defective GSIS in β-MDM2KO mice. Eighteen-week-old male β-MDM2KO mice (KO), β-MDM2–p53 DKO mice and its WT littermates were used. (**a**) Isolated islets were subjected to immunoblotting using an anti-MDM2, -p53 or -β-actin antibody. Representative immunoblot images from three independent experiments are shown. (**b**) GTT. (**c**) Serum insulin levels during the GTT in **b**. (**d**) ITT. (**e**) Static GSIS in the isolated islets as in Fig. 1g. Note that insulin content in the islets was similar among the three groups. (**f**) Glucose (20 mM)-stimulated intracellular ATP production in the isolated islets. The values are expressed as fold change over basal level (2.8 mM glucose). *P<0.05 (KO versus WT, *n*=5), ^#^*P*<0.05 (KO versus DKO, *n*=5) (Student's *t*-test). All data are represented as the mean±s.e.m.

To further confirm whether p53 activation is responsible for glucose intolerance and defective GSIS in β-MDM2KO mice, we generated β-cell-specific MDM2–p53 double KO (DKO) mice by crossing β-MDM2KO mice with *p53*^*floxed/floxed*^ mice^20^. Augmented expression of p53 by deletion of *MDM2* was abolished in islets of DKO mice (Fig. 4a). Glucose intolerance and defective GSIS in β-MDM2KO mice were largely reversed by concomitant genetic deletion of p53, whereas insulin sensitivity was comparable among the three groups (Fig. 4b–e and Supplementary Fig. 3).

### MDM2 deficiency causes mitochondrial dysfunctions in β-cells

**Figure 5:**
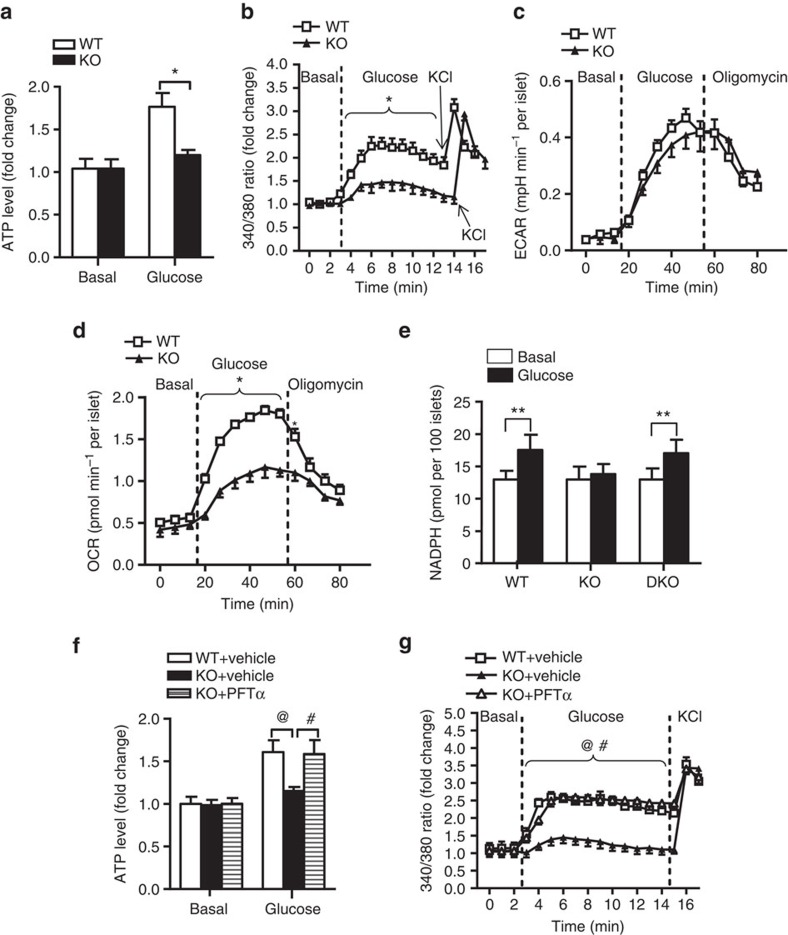
Inhibition of MDM2 impairs mitochondrial metabolism in pancreatic β-cells in p53-dependent manner. Pancreatic islets isolated from 10-week-old male β-MDM2KO mice, DKO and its WT littermates were used. Intracellular levels of ATP (**a**), calcium influx (**b**), ECAR (**c**) and OCR (**d**) in the isolated islets in response to various stimuli as indicated (**a**,**b**: *n*=4; **c**,**d**: *n*=5). (**e**) Intracellular NADPH levels measured at 30 min after stimulation with glucose (20 mM) in the isolated islets. (**f**,**g**) The isolated islets treated with PFTα (10 μM) or DMSO as vehicle were subjected to assessment of ATP production (**f**) and calcium influx (**g**) (*n*=4). *P<0.05 (WT versus KO), ^@^*P*<0.05 (WT+vehicle versus KO-vehicle), ^#^*P*<0.05 (KO-vehicle versus KO-PFTα), **P<0.05 (Student's *t*-test). All data are represented as the mean±s.e.m.

The MDM2–p53 pathway has been implicated in both cell apoptosis and survival^21^. We therefore examined the morphology of pancreatic islets of β-MDM2KO mice and WT controls. The β-cell mass, pancreatic insulin content and distribution of α- and β-cells in islets were comparable between β-MDM2KO mice and WT littermates (Supplementary Fig. 5a–e). There was no obvious difference in the number of total and docked insulin granules between β-MDM2KO mice and WT littermates (Supplementary Fig. 5f–h). The expression levels of key genes involved in insulin biosynthesis (pancreatic and duodenal homeobox 1 (*PDX1*) and insulin 2 (*INS2*)), glucose uptake (glucose transporter 2 (*GLUT2*)), glucose metabolism (glucose kinase (*GCK*) and pyruvate dehydrogenase kinase 1 (*PDH*)) were also similar between the two groups (Supplementary Fig. 5i). Electron microscopic analysis showed no obvious difference in morphology, number and area of mitochondria between β-MDM2KO mice and WT controls (Supplementary Fig. 5j–l). On the contrary, glucose-stimulated ATP production and calcium influx were significantly abrogated in MDM2-deficient islets (Fig. 5a,b) or β-cells treated with nutlin-3a (Supplementary Fig. 6a,b). Furthermore, increase in ATP/ADP ratio by glucose stimulation in pancreatic islets was abrogated by *MDM2* deletion (Supplementary Fig. 7)

**Figure 6:**
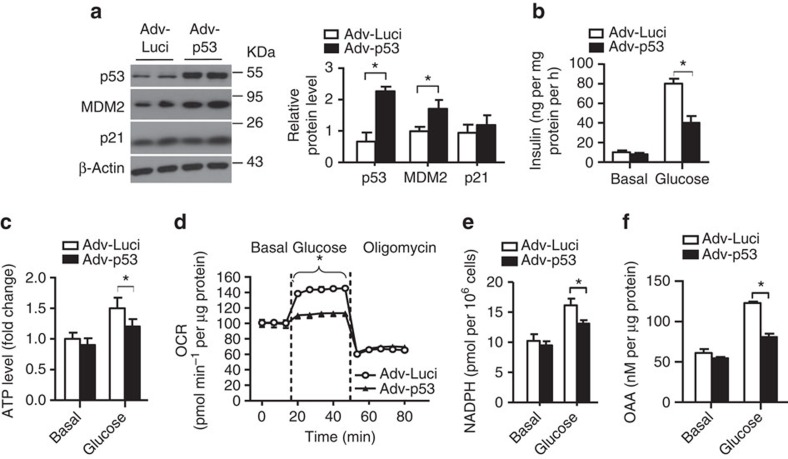
Adenovirus-mediated overexpression of p53 attenuates GSIS and mitochondrial functions. MIN6 β-cells were infected with adenovirus encoding luciferase (Adv-Luci) or p53 (Adv-p53) at m.o.i.=50 for 24 h, followed by serum and glucose starvation for an hour. (**a**) Representative images of immunoblotting for p53, MDM2, p21 and β-actin from three independent experiments are shown. The right panel is the densitometric analysis for the relative abundance of p53, MDM2 and p21 normalized with β-actin. (**b**) Static insulin secretion in response to glucose (20 mM) stimulation. Note that there was no obvious difference in insulin content in MIN6 cells infected with Adv-Luci or Adv-p53 for 24 h. (**c**) Intracellular ATP levels measured at 10 min after stimulation with glucose (20 mM). (**d**) OCR was measured under basal, glucose (20 mM)- and oligomycin (5 μM)-stimulated conditions at indicated time points. (**e**) NADPDH and (**f**) OAA production measured at 30 min after stimulation with glucose (20 mM). *P<0.05 (*n*=4) (Student's *t*-test). All data are represented as the mean±s.e.m.

We next examined the impacts of MDM2 deficiency on β-cell metabolism by measuring oxygen consumption rate (OCR; an indicator of aerobic metabolism of glucose via TCA cycle and mitochondrial oxidative phosphorylation), as well as extracellular acidification rate (ECAR; a parameter of glycolytic flux), with the XF24 extracellular flux analyser. Genetic deletion or pharmacological inhibition of MDM2 had no effect on the ECAR level, but significantly abolished glucose-induced OCR (Fig. 5c,d and Supplementary Fig. 6c,d). The reduced OCR in β-cells with MDM2 inactivation was associated with a drastic reduction of glucose-stimulated production of NADH and NADPH, two important intermediates derived from glucose metabolism in TCA cycle (Fig.5e and Supplementary Fig. 6e,f). Likewise, adenovirus-mediated overexpression of p53 resulted in a significant reduction of insulin secretion, ATP and NADPH production and OCR in response to glucose stimulation (Fig. 6a–e). Noticeably, short-term overexpression of p53 by ∼2.4-fold had no obvious effect on apoptosis and proliferation in MIN6 cells (Supplementary Fig. 8). On the other hand, reductions in ATP and NADPH levels and calcium influx in response to glucose stimulation in MDM2-null islets were reversed by inhibition of p53 (Figs 4f and 5e–g). Taken together, these data suggest that disruption of the MDM2–p53 axis impairs TCA cycle in β-cells, thereby leading to defective GSIS.

### Restoration of PC rescues MDM2-null phenotypes in β-cells

**Figure 7:**
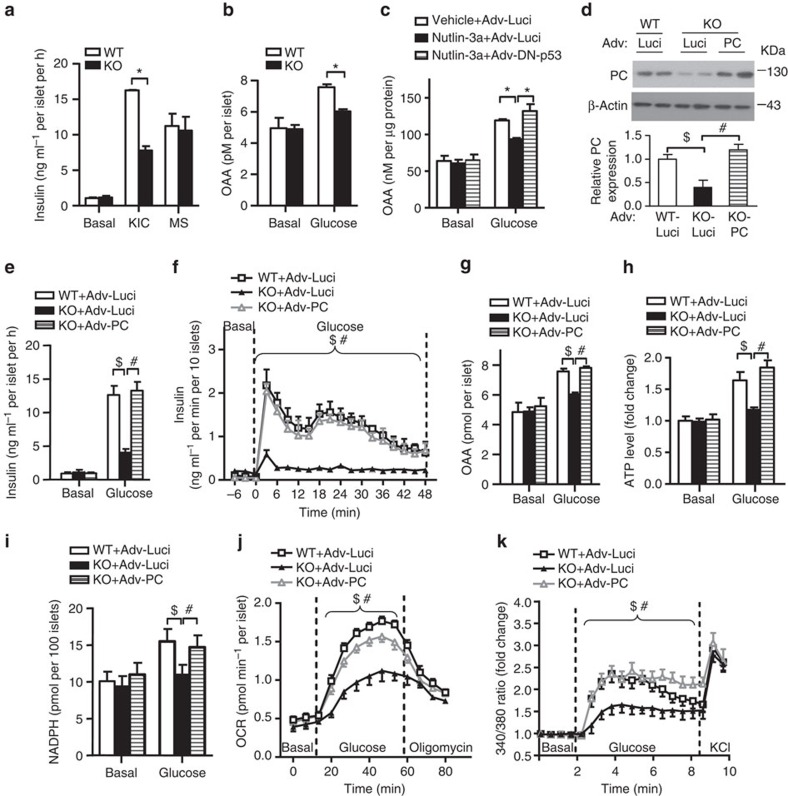
PC mediates the regulatory effects of MDM2 on GSIS and mitochondrial metabolism. (**a**) Pancreatic islets isolated from 10-week-old male β-MDM2KO mice and its WT littermates were stimulated with non-glucose secretagogues, including α-ketoisocaproic acid (KIC, 10 mM), and methyl succinate (MS, 10 mM) for 30 min, followed by measurement of insulin levels in the conditional medium (*n*=5). (**b**) Intracellular OAA levels in response to glucose stimulation (20 mM) for 30 min (*n*=4). (**c**) MIN6 β-cells were infected with adenovirus encoding luciferase (Adv-Luci) or dominant negative form of p53 (Adv-DN-p53) at m.o.i.=50 for 24 h. The infected cells were treated with nutlin-3a (10 μg ml^−1^) or DMSO as vehicle for 6 h. The cells were subjected to serum and glucose starvation for an hour. Intracellular OAA levels were measured after glucose (20 mM) stimulation for 30 min (*n*=4). (**d**–**k**) The islets isolated from 10-week-old male β-MDM2KO mice and its WT littermates were infected with adenovirus encoding luciferase (Adv-Luci) or pyruvate carboxylase (Adv-PC) at m.o.i.=100 for 24 h. (**d**) The infected islets were subjected to immunoblotting using an antibody against PC or β-actin as indicated. Representative immunoblot images from three independent experiments are shown. The bottom panel is the densitometric analysis for the relative abundance of PC normalized with β-actin (*n*=4). Static (**e**) or dynamic (**f**) GSIS (*n*=8). Note that insulin content of isolated islets was similar among the three groups. Intracellular levels of OAA (**g**) and ATP (**h**) were measured on glucose stimulation for 30 and 10 min, respectively. (**i**) NADPH levels were measured on glucose stimulation for 30 min. OCR (**j**) and calcium influx (**k**) were measured as in Fig. 5 (*n*=4). *P<0.05, ^$^*P*<0.05 (WT+Adv-Luci versus KO+Adv-Luci), ^#^*P*<0.01 (KO+Adv-Luci versus KO+Adv-PC) (Student's *t*-test). All data are represented as the mean±s.e.m.

To identify the mechanism whereby MDM2 deficiency causes impaired TCA cycle, we stimulated the islets with the mitochondrial substrates α-ketoisocaproic acid, which can be directly metabolized to acetyl-CoA in mitochondria, and methyl succinate, which directly activates the complex II, supplies succinate and then converts to oxaloacetate (OAA) in the TCA cycle (Supplementary Fig. 9a). α-ketoisocaproic acid- but not methyl succinate-induced insulin secretion and calcium influx was impaired in MDM2-null islets when compared with its WT controls (Fig. 7a and Supplementary Fig. 9b,c). Similar result was also observed in MIN6 β-cells treated with nutlin-3a (Supplementary Fig. 9d), suggesting that PC-mediated production of OAA in the TCA cycle could be impaired in β-cells, with inhibition of MDM2. To test this hypothesis, we first measured OAA level in islets and β-cells on modulation of MDM2 and p53 levels. Glucose-stimulated OAA production was reduced in MDM2-deficient islets and MIN6 β-cells treated with nutlin-3a or with overexpression of p53 when compared with their corresponding control groups, and was reversed by inactivation of p53 (Figs 6f and 7b,c). We then measured the expression of mitochondrial enzymes that can alter OAA production in TCA cycle. Expression of PC was reduced in isolated islets but not in hypothalamus of β-MDM2KO mice (Supplementary Figs 1b and 10a,b), whereas the expression of malic enzyme 1 or 2 in the isolated islets was indistinguishable between the two groups (Supplementary Fig. 10a). Likewise, the mRNA level of *PC* was decreased in MIN6 β-cells treated with nutlin-3a when compared with vehicle control (Supplementary Fig. 10c). The reduction in PC expression was reversed by inhibiting p53 with PFTα in MDM2-deficient islets, suggesting that MDM2 regulates PC expression via p53 (Supplementary Fig. 10d).

To test whether replenishment of PC could reverse the defects in MDM2-null islets, islets from β-MDM2KO mice and WT controls were infected with adenovirus encoding luciferase or PC (Fig. 7d). PC levels in MDM2-null islets infected with adenovirus encoding PC was restored to a level similar to its WT controls (Fig. 7d). Notably, defects in GSIS, glucose-mediated production of OAA, ATP and NADPH, mitochondrial respiration, as well as calcium influx in MDM2-null islets were all rectified by restoration of PC expression when compared with MDM2-null islets infected with luciferase control (Fig. 7e–k). These data support the notion that lowered PC expression is responsible for the abrogated GSIS on p53 activation.

### p53 directly suppresses transcription of *PC* gene in β-cells

**Figure 8:**
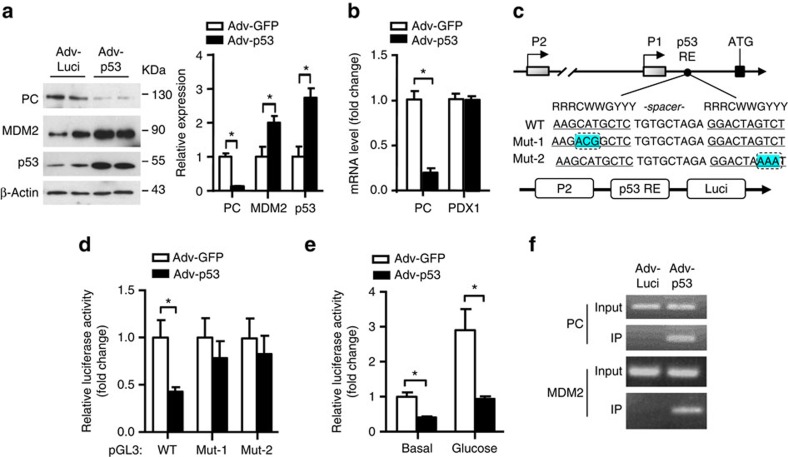
p53 suppresses the transcriptional activation of the mitochondrial enzyme PC in β-cells. (**a**,**b**) MIN6 cells were infected with adenovirus encoding luciferase (Adv-Luci) or p53 (Adv-p53) at m.o.i.=50 for 24 h, followed by serum starvation for 6 h. (**a**) Immunoblotting (left panel) and the densitometric analysis (right panel) for PC, MDM2, p53 or β-actin (*n*=4). (**b**) Relative mRNA abundance of the *PC* and *PDX1* genes (*n*=4). (**c**) Schematic diagram showing the structure of the mouse *PC* gene, which is controlled by the proximal (P1) promoter and the distal (P2) promoter (upper panel). Putative p53 RE in the promoter region of *PC* gene is identified by matching with the p53 RE consensus sequence (2 decamer motif RRRCWWGYYY separated by a spacer of 0–13 base pair, where R is A or G, W is A or T, and Y is C or T). The two decamers are underlined and mutations created in the p53 RE are highlighted with blue. The bottom panel showed the constructs containing the P2 promoter and the p53 RE (WT) or its mutants (Mut-1 and Mut-2) for luciferase assay. (**d**–**f**) MIN6 cells were transfected with luciferase reporter constructs, followed by infection with Adv-GFP or Adv-p53at m.o.i.=50 for 24 h. (**d**) Measurement of luciferase activity (*n*=4). (**e**) The cells were fasted in glucose-free medium for 1 h and then stimulated with glucose (20 mM) for 12 h before measurement of luciferase activity (*n*=4). (**f**) MIN6 cells were infected with Adv-Luci or Adv-p53, followed by chromatin immunoprecipitation using an anti-FLAG antibody. The immunocomplex were subjected to PCR amplification using specific primers against the promoter region of *PC* and *MDM2* consisting of p53 RE (*n*=4). Representative immunoblots and DNA gel images from three independent experiment are shown. *P<0.05 (Student's *t*-test). All data are represented as the mean±s.e.m.

To test whether p53 directly regulates expression of *PC*, we overexpressed WT p53 in MIN6 β-cells by adenoviral gene transfer system. This analysis demonstrated that p53 suppressed both protein and mRNA levels of PC (Fig. 8a,b). Through *in silico* analysis, we identified a putative p53 response element (RE) between −6,810 and −6,781 bp relative to the start codon in the promoter region of the *PC* gene (Fig. 8c). To investigate whether p53 regulates the promoter activity of *PC* via this potential p53 RE, we cloned the promoter region containing the p53 RE and the distal promoter (P2, between −92,280 and −91,186 bp) (which is responsible for glucose-mediated PC expression in pancreatic β-cells^22^) of the *PC* gene into the luciferase reporter vector pGL3 (Fig. 8c). Overexpression of p53 significantly inhibited the promoter activity of *PC* in β-cells when compared with those overexpressing green fluorescent protein (GFP) controls (Fig. 8d).

To further confirm that p53 regulates *PC* promoter activity via the p53 RE, we generated two mutant constructs in which the CAT at the first central CWWG (where W is A or T) and the GTC at the second decamer motif were changed to ACG and AAA through site-specific mutagenesis, respectively (Fig. 8c,d). The inhibitory effect of p53 on P2 promoter activity was lost in the mutants (Fig. 8d). In addition, induction of the *PC* promoter activity by glucose was abolished by overexpression of p53 (Fig. 8e). Chromatin immunoprecipitation analysis showed that there was a significant enrichment of p53 to the p53 RE in the *PC* promoter (Fig. 8f). In addition, the immunoprecipitated complex also contained the promoter of *MDM2* (Fig. 8f), which contains the p53 RE^23^. Collectively, these results indicated that p53 downregulates PC expression by directly interacting with the p53 RE in the *PC* gene, thereby leading to transcriptional repression.

### Inhibition of p53 relieves β-cell dysfunction in diabetes

**Figure 9:**
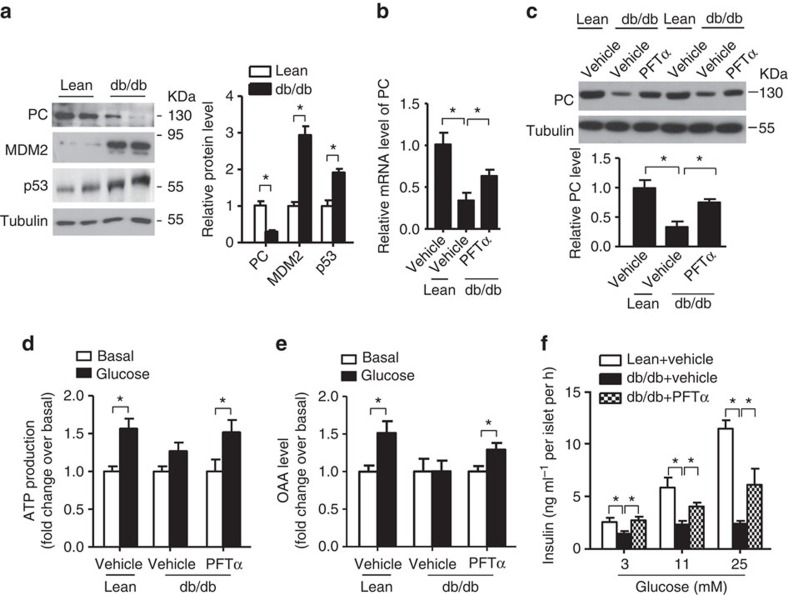
Inactivation of p53 alleviates defective GSIS in diabetic mice. (**a**) Immunoblotting analysis of PC, p53, MDM2 and β-tubulin in the islets isolated from 16-week-old male db/db diabetic mice and their lean controls. The right panel is the densitometric analysis for relative abundance of PC, MDM2 and p53 normalized with β-tubulin (*n*=4). (**b**,**c**) Isolated islets from db/db diabetic mice and its lean controls were treated with PFTα (10 μm) or DMSO as vehicle control for 24 h, followed by QPCR (**b**) and immunoblotting (**c**) analysis of PC (*n*=4). (**d**,**e**) Intracellular levels of ATP and OAA in treated islets were measured at 10 and 30 min after glucose stimulation (20 mM), respectively (*n*=4). (**f**) Static GSIS in the treated islets were measured at 30 min after glucose stimulation (*n*=5). Note that insulin content of the isolated islets was comparable between different treatment groups. *P<0.05 (Student's *t*-test). All experiments were repeated for at least three times and representative immunoblots are shown. All data are represented as the mean±s.e.m.

To test whether aberrant MDM2–p53–PC signalling axis correlates with impaired GSIS in islets of db/db diabetic mice, we quantified protein expression of MDM2, p53 and PC by immunoblotting. Protein expression of PC was decreased while p53 and MDM2 were increased in diabetic islets when compared with those isolated from WT healthy controls (Fig. 9a). Of note, the increase in MDM2 indicates an upregulation of p53 activity. Importantly, treatment with the p53 inhibitor PFTα ameliorated defective GSIS in diabetic islets, and this improvement was linked to increased PC expression and the partial restoration of ATP and OAA production in response to glucose stimulation (Fig. 9b–f).

**Figure 10:**
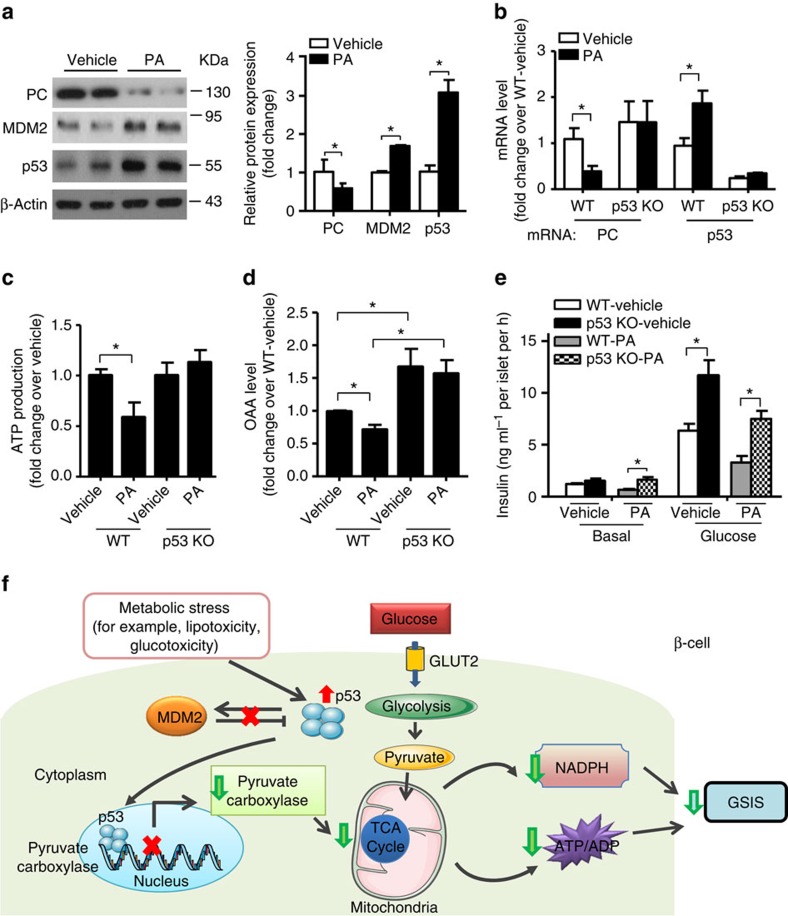
β-cell-specific ablation of p53 alleviates palmitic acid-induced impairment of mitochondrial metabolism and GSIS. (**a**) MIN6 β-cells were treated with palmitic acid (PA, 500 μM) for 24 h, followed by immunoblotting with an antibody against MDM2, p53, PC or β-actin as indicated. The right panel is the densitometric analysis for the relative abundance of MDM2, p53 and PC normalized with β-actin (*n*=4). (**b**–**e**) Pancreatic islets were isolated from 14-week-old male β-cell-specific p53 KO mice and its WT controls, followed by treatment with PA (500 μM) for 24 h. (**b**) mRNA level of *PC* and *p53* in the treated islets (*n*=3). (**c**,**d**) Intracellular levels of ATP (**c**) and OAA (**d**) in the islets with glucose stimulation (20 mM) for 10 and 30 min, respectively. (**c**) The value is normalized with their respective vehicle-treated control with glucose stimulation (*n*=4). (**d**) The value is normalized with WT-vehicle with glucose stimulation (*n*=4). (**e**) Static GSIS assay measured at 30 min after glucose stimulation (*n*=5). *P<0.05 (Student's *t*-test (**a**,**b**,**c**,**e**), one-way analysis of variance with Bonferroni correction for multiple comparisons (**e**)). All data are represented as the mean±s.e.m. Note that insulin content of the isolated islets was comparable between different treatment groups. (**f**) Proposed model for the role of MDM2–p53–PC signalling axis in GSIS. Sustained elevation of p53 or dysregulation of the MDM2–p53 signalling axis elicited by a wide variety of chronic stress signals directly represses expression of PC by binding to the promoter region of *PC* via the p53 RE. Reduction of PC impairs TCA cycle, leading to decreased production of ATP, OAA and amplifying factors (such as NADPH), resulting in defective GSIS.

Elevated levels of circulating free fatty acid (FFA) are known to be associated with type 2 diabetes, and chronic exposure of FFA impairs GSIS and mitochondrial metabolism in pancreatic islets^3^. Similar to the islets isolated from db/db mice, expression of PC was reduced while MDM2 and p53 were elevated in MIN6 β-cells treated with palmitic acid when compared with the cells treated with vehicle control (Fig. 10a). To determine whether the deteriorative effects of palmitic acid on β-cell functions are mediated by p53, we generated β-cell-specific p53 KO mice by crossing *p53*^*floxed/floxed*^ mice with RIP-Cre transgenic mice. β-cell-specific p53 KO mice displayed a mild increase in β-cell mass when compared with its WT control, despite that the difference did not reach statistical significance (Supplementary Fig. 11). Palmitic acid markedly reduced the expression of *PC* mRNA but induced the expression of *p53* mRNA in WT islets when compared with those treated with vehicle control (Fig. 10b). In contrast, expression levels of PC and p53 were indistinguishable between vehicle- and palmitic acid-treated groups in p53-deficient islets (Fig. 10b). As expected, palmitic acid strongly inhibited glucose-stimulated intracellular production of ATP and OAA, as well as insulin secretion, whereas genetic ablation of *p53* in β-cell was able to alleviate these suppressive effects of palmitic acid on β-cell functions, at least in part, by restoring mitochondrial metabolism (Fig. 10c–e).

## Discussion

Defective insulin secretion exemplified by impaired GSIS is a hallmark of type 2 diabetes. Mitochondria dysfunction in β-cells has been postulated as an important culprit for the defective insulin secretion. However, the underlying mechanisms remain elusive. In this study, we identified the MDM2–p53–PC axis as a key player that couples mitochondrial metabolites to GSIS. The aberrant activity of this axis is an important contributor to mitochondrial dysfunction and defective GSIS in β-cells, thereby leading to type 2 diabetes.

Mitochondria act as a nutrient sensor and metabolite generator connecting glucose metabolism to insulin secretion in β-cells^2^. PC has been proposed as a key player in coupling mitochondrial metabolism to first- and second-phase insulin secretion. PC-mediated carboxylation of pyruvate not only provides an anaplerotic input for the TCA cycle and subsequent production of ATP by supplying OAA but also contributes to the formation of NADPH, an important amplifying factor for GSIS^24^,^25^. Mass isotopomer multi-ordinate spectral analysis revealed that PC flux in mitochondria is markedly induced in response to glucose stimulation in pancreatic β-cells, which is closely associated with GSIS^26^. Inhibition of PC abolishes anaplerosis of the TCA cycle and ATP production, resulting in impaired GSIS in β-cells, whereas overexpression of PC exerts opposite effects^24^,^27^. PC mediates the protective effects of geniposide and PPAR-α overexpression against defective GSIS in INS-1E cells challenged with glucotoxicity and lipotoxicity, respectively^28^,^29^. Such protective effect of PC may be due to its indispensable role in maintaining the balance of intermediates of glucose metabolism in the TCA cycle^28^. Knockdown of PC or inhibition of PC activity by phenylacetic acid has been shown to potentiate the detrimental effects of glucotoxicity and lipotoxicity on β-cell functions^30^. A genetic linkage study in diabetic patients has identified a significant association of a single-nucleotide polymorphism in the *PC* gene with the magnitude of acute insulin secretion^31^. Furthermore, a markedly reduced expression of PC has been observed in pancreatic islets of both animal models and patients with type 2 diabetes^32^,^33^. However, the molecular pathways and pathophysiological relevance of PC downregulation in diabetic β-cells remain unknown. Our present study identified MDM2 and p53 as a Ying–Yang pair to fine tune the expression of PC in pancreatic β-cells, thereby regulating TCA cycle, ensuing ATP production and insulin secretion. Notably, glucose not only acutely induces PC expression but also promotes MDM2–p53 interaction^34^,^35^, suggesting that the MDM2–p53–PC axis is an integral component mediating GSIS. Our results also demonstrated that diminished PC expression, and impaired mitochondrial function and GSIS in the pancreatic islets of type 2 diabetic mice are attributed to the imbalance between MDM2 and p53. Furthermore, defective GSIS in the diabetic islets and MDM2-deficient islets can be reversed by replenishment with PC, further supporting the role of PC as an obligatory downstream target of MDM2–p53 in coupling glucose metabolism, TCA cycle and insulin secretion. In line with our results, inactivation of p53 by genetic deletion or pharmacological inhibitor has been shown to improve glucose tolerance by enhancing mitochondrial respiration, ATP production and GSIS in mouse models with type 1 and type 2 diabetic, both of which display defective GSIS at the early stage^12^.

In both rodents and human, *PC* expression in different tissues is controlled by two alternative promoters under different physiological circumstances, including the proximal promoter activity in liver and adipose tissue, and distal promoter activity in pancreatic β-cells^36^,^37^,^38^. Several pancreatic islet-specific transcription factors, such as PDX1 and forkhead box protein A2, induce *PC* expression in a β-cell-specific manner^36^,^37^,^38^. Three canonical E-boxes in the distal promoter act as a glucose RE to mediate the stimulatory effect of glucose on *PC* expression in β-cells. The canonical p53 RE identified in our present study is located within the promoter of the *PC* gene. Furthermore, glucose-stimulated distal promoter activity of *PC* is largely abrogated by overexpression of p53 in insulin-secreting cells, whereas mutation of the p53 RE abolishes the suppressive effects of p53 on *PC* expression. Notably, overexpression of p53 has no obvious effect on distal promoter activity of *PC* in HEK293 cells (Supplementary Fig. 12), indicating that p53 may interact with other β-cell-specific transcription co-factor(s) to repress *PC* expression. On the other hand, although p53 has been shown to modulate glycolytic and respiratory pathways via induction of mitochondrial cytochrome c oxidase in several cancer cell lines^5^, we did not observe such effects of p53 or MDM2 in pancreatic β-cells, suggesting that the metabolic regulation of p53 is highly cell type-specific.

Despite the crucial role of p53 in apoptosis and growth arrest^21^, we showed that β-cell activation of p53 by genetic or pharmacological inhibition of MDM2 has no obvious impact on β-cell mass or death in lean mice (Supplementary Fig. 5a,b). Apart from MDM2, p53 level is negatively regulated by several other E3 ubiquitin ligases including MDM4, arf-bp1, Pirh2 and COP1, via ubiquitination in an MDM2-independent manner^39^. Indeed, the differential effects of p53-mediated stress responses are controlled by distinct E3 ubiquitin ligases^39^. For example, β-cell deletion of arf-bp1 leads to overt β-cell loss and diabetes with ageing, and such phenotypes can be reversed by co-deletion of p53 (ref. 40). Whether MDM2 and other p53 inhibitors play a redundant or distinct role in the regulation of β-cell mass or function is currently unknown. Furthermore, it has been reported that activation of the apoptotic targets of p53 requires higher levels of p53, perhaps due to lower p53-binding affinity in the apoptotic targets^41^. Consistent with this notion, a marked elevation of p53 activity by either transgenic overexpression of its short isoform or its upstream positive regulator (such as microRNA-200) induces β-cell apoptosis^14^,^42^. In contrast, our study showed that deletion of *MDM2* only induces a modest induction of p53 in β-cells (∼2.1-fold), which may be insufficient to induce the apoptotic gene expression. Therefore, it is likely that a modest increase of p53 impairs GSIS, whereas a higher level of p53 induces β-cell apoptosis. On the basis of these observations, we propose a differential role of p53 at different stages of type 2 diabetes: p53 expression in pancreatic islets can be induced by various metabolic insults (such as lipotoxicity), endoplasmic reticulum and oxidative stresses. At the early phase of the disease, a modest increase of p53 inhibits GSIS by repressing PC expression in pancreatic β-cells. However, with the progression of the disease, p53 is further elevated, which in turn triggers β-cell apoptosis, leading to overt hyperglycaemia.

p53 in pancreatic β-cells is activated by a number of stress signals, including lipotoxicity, glucotoxicity, pro-inflammatory cytokines and DNA damage agents^11^,^12^,^13^,^43^. In line with these findings, we found that expression of p53 is increased in db/db diabetic islets or in islets challenged with toxic lipids. Furthermore, several intracellular factors and miRNAs (such as miRNA200c and transcription factor T-cell factor 7-like 2 (TCF7L2)) has been shown to be a contributor to elevated p53 expression in β-cells^42^,^44^. Nevertheless, how p53 is activated by different stress signals and whether this p53 activation is mediated via MDM2 or other upstream regulators such as TCF7L2 in β-cells need further investigations.

In summary, our findings uncovered the MDM2–p53 pathway as a key molecular connection between mitochondrial metabolism and insulin secretion by regulating the expression of PC, which controls the homeostasis of intermediary metabolites in the TCA cycle (Fig. 10f). Dysfunctional MDM2–p53–PC axis is an important mediator for defective GSIS in type 2 diabetes, and pharmacological interventions targeting this signalling axis, such as inhibition of p53 activity, may be effective for glycaemia control in diabetes. On the other hand, given that high levels of PC and MDM2 and/or reduction of p53 promote tumour growth, the MDM2–p53–PC axis may also act as a key player in linking mitochondrial metabolism with cancer.

## Methods

### Materials and cell culture

Rabbit monoclonal antibody against β-tubulin (Catalogue #2128, 1:2,500 for WB) and mouse monoclonal antibody against p53 (Catalogue #2524, 1:1,000 for WB) were obtained from Cell Signaling Technology. Rabbit monoclonal antibodies against and PC (Catalogue #Ab128952, 1:1,000 for WB), and rabbit polyclonal antibody against vesicle-associated membrane protein 2 (VAMP2; Catalogue #ab3347, 1:100 for immunohistochemical (IHC) and 1:2,000 for WB) and p21 (Catalogue #ab7960, 1:1,000 for WB) were purchased from Abcam. Mouse monoclonal antibody against MDM2 (Catalogue #04-1530, 1:50 for IHC and 1:500 for WB) was from Merck Millipore. Goat polyclonal antibody against β-actin (Catalogue #sc-1616, 1:2,000 for WB) was from Santa Cruz Biotechnology. Mouse monoclonal anti-FLAG antibody (Catalogue #F1804, 1:200 for IHC and 1:5,000 for WB), rabbit polyclonal antibody against glucagon (Catalogue #SAB4501137, 1:100 for IHC), palmitic acid, fatty acid free-bovine serum albumin (BSA), L-arginine, nutlin-3a and PFTα were from Sigma. Mouse monoclonal antibody against insulin (Catalogue #2IP10-D6C4, 1:200 for IHC) was from HyTest. Collagenase P was obtained from Roche Life Science. Human recombinant insulin was from Novo Nordisk. Mouse insulinoma MIN6 cells (a kind gift from Junichi Miyazaki Laboratory, Osaka University) and HEK293 cells (American Type Culture Collection) were maintained in DMEM supplemented with 10% fetal bovine serum (FBS; Life Technologies) and free of mycoplasma contamination.

### Animal studies

All animals were sex- and age-matched, and littermates were used, as indicated in the figure legends. Animals were allocated to their experimental group according to their genotypes, and therefore no randomization is used, unless otherwise noted. The investigators were not blinded to the experimental groups. Homozygous MDM2 floxed mice (*MDM2*^*floxed/floxed*^), obtained from Professor Guillermina Lozano (MD Anderson Cancer Center, The University of Texas)^45^, were crossed with RIP-Cre mice (*RIP-Cre*^*+*^*-MDM2*^−/*−*^) to generate heterozygous RIP-Cre-MDM2 floxed mice (*RIP-Cre*^*+*^*MDM2*^*floxed/*−^) and *MDM2*^*floxed/−*^ mice. *RIP-Cre*^*+*^*MDM2*^*floxed/−*^ mice were intercrossed with *MDM2*^*floxed/−*^ mice to generate RIP-Cre control mice (*RIP-Cre*^*+*^*-MDM2*^−/*−*^), WT littermates (*MDM2*^*floxed/floxed*^) and β-MDM2KO (*RIP-Cre*^*+*^*MDM2*^*floxed/floxed*^) mice. To generate β-cell-specific MDM2–p53 DKO mice, β-MDM2KO mice were crossed with *p53*^*floxed/floxed*^ mice (Jackson Laboratory)^20^. *p53*^*floxed/floxed*^ mice were crossed with RIP-Cre mice to generate β-cell-specific p53 KO (*RIP-Cre*^*+*^-*p53*^*floxed/floxed*^) mice and its WT littermates (*p53*^*floxed/floxed*^). All the above mice were crossed with C57 BL6/J mice for eight generations and maintained on the C57 BL6/J genetic background. The mice had free access to food and water and were kept in cages in a 12-h light/dark cycle and were fed with a standard chow (Purina) with 20% kcal from protein, 10% kcal from fat and 70% kcal from combined simple carbohydrates. Glucose tolerance test (GTT) and ITT were performed in 6-h fast mice^46^. Briefly, the fasted mice were intraperitoneally injected with glucose (2 g kg^−1^) or insulin (0.5 U kg^−1^) for GTT and ITT, respectively. Insulin secretion in response to intraperitoneal injection of glucose (2 g kg^−1^) or L-arginine (1 g kg^−1^) was performed in the 6-h-fasted mice^46^. Blood samples were taken from the tail vein for the measurement of glucose and insulin levels using a glucose meter and an insulin ELISA kit (Catalogue #32380, Antibody and Immunoassay Services, The University of Hong Kong), respectively.

Twelve-week-old male C57BL6/N mice (purchased from The Laboratory Animal Unit of The University of Hong Kong) were randomly assigned to nutlin-3a treatment and dimethylsulphoxide (DMSO) control groups. The mice were administrated with nutlin-3a (10 mg kg^−1^) or DMSO by oral gavages, followed by fasting for 6 h. The mice were then subjected to GTT, ITT and insulin secretory experiments as above. Sixteen-week-old male db/db diabetic mice and its lean controls were from Jackson Laboratory and maintained on the C57BLKS genetic background. All animal experimental protocols were approved by the Animal Ethics Committee of The University of Hong Kong.

### Islet isolation and insulin secretion assay

The mice were fasted for 4 h and killed by cervical dislocation. Pancreatic islets and exocrine cells were isolated as previously described^46^. Briefly, pancreas was perfused with collagenase P (1.4 mg ml^−1^) via bile common ducts, followed by digestion at 37 °C for 10 min. The digested pancreas was filtered with a 500 and a 70-μm cell strainer. Captured islets were washed with solution G (HBBS (Gibco) with 0.1% BSA), and then picked manually under a microscope and cultured in RPMI 1640 with 10% FBS overnight. The isolated islets with similar size were subjected to assessment for static and dynamic insulin secretion^46^. Briefly, isolated islets were washed twice with Krebs Ringer bicarbonate (KRB) buffer containing 0.1% fatty acid-free BSA supplemented with 2.8 mM glucose for 1 h, followed by treatment with different stimulants for various time periods as specified in each figure legend. For the perfusion experiment, the isolated islets were incubated with KRB buffer for 30 min and perfused with KRB buffer containing 2.8 mM glucose for 6 min, and the perfusate was then switched to KRB buffer containing 10 mM glucose. Eluted fractions were collected at 3-min intervals for 48 min. The first- and second- phase insulin secretion was defined as 0–10 and 10–48 min, respectively, in islets on glucose stimulation, according to previous publications^46^,^47^. Insulin secreted in each fraction was measured using the insulin ELISA kit and normalized by the number of islets.

### Measurement of insulin content in pancreas and islets

Pancreas or isolated islets were incubated with acid-ethanol (1.5% HCl in 70% ethanol) at −20 °C overnight, followed by homogenization. The homogenized lysate was incubated at −20 °C overnight and centrifuged at 10,000*g* at 4 °C. The pancreatic extract was neutralized with Tris-buffer (pH 7.5), followed by measurement of insulin with the mouse insulin ELISA.

### Quantitative real-time PCR

Total RNA was extracted from isolated islets or MIN6 cells using TRIzol reagent (Life Technologies), and cDNA was synthesized from 0.5 μg total RNA by reverse transcription using an ImProm-II reverse transcription kit (Promega) with random hexamer primers. Quantitative real-time PCR was performed using SYBR Green QPCR system (Qiagen) with specific primers (Supplementary Table 2). The PCR reactions were performed using StepOnePlus Real-Time PCR system (Applied Biosystems). The mRNA level of target gene expression was normalized against the average value of GAPDH and β-actin.

### Generation and purification of adenovirus and AAV

To generate adenoviral vectors for the expression of p53 or PC, cDNA encoding full-length mouse *p53* or human *PC* was cloned into pshuttle-CMV vector, and then subcloned into pAdeasy-1 adenoviral backbone vector (Stratagene) via recombination in BJ5183 competent cells^48^. The positive clones were identified by digestion with the restriction enzyme PacI and then confirmed by DNA sequencing. To construct adenoviral vector encoding DN-p53, PCR-based site-directed mutagenesis was performed to introduce R172H mutation in mouse *p53* using QuikChange II XL Site-Directed Mutagenesis Kit with the mutagenic primers (Supplementary Table 2)^49^. Adenoviruses encoding luciferase were generated in our previous study^48^. Adenovirus encoding GFP was obtained from Vector Biolabs. The adenoviruses were purified using Vivapure AdenoPACK 100 RT VS-AVPQ102 (Sartorius) according to the manufacturer's instruction. The titre of adenovirus was determined by end-point dilution titration in HEK293 cells. Isolated islets with similar size were selected and cultured overnight for adenovirus infection^46^. The islets were infected with the adenovirus at 100 multiplicity of infection (m.o.i.) for 4 h at 37 °C (assuming 1,000 cells per islet on average) followed by incubation with fresh medium for 18 h. MIN6 β-cells were infected with the adenovirus at 50 m.o.i. and cultured as above.

AAV vector encoding GFP under the control of modified mouse insulin promoter was generated as previously described^19^. To generate AAV vector encoding DN-p53, cDNA encoding mouse *p53*^*R172H*^ was amplified and cloned from the pshuttle-DN-p53 vector, and the (BspHI–BglII) PCR fragment was inserted into the AAV vector with mouse insulin promoter. To generate recombinant AAV particles, HEK293T cells were co-transfected with the AAV vector, pRep2Cap8 vector and the helper vector using the helper-free method. On the third day after transfection, the cells were lysed in a lysis buffer (50 mM Tris Cl, 150 mM NaCl, 2 mM MgCl_2_, pH 8.0) by freeze and thaw cycle for three times, followed by polyethylene glycol/aqueous two-phase partitioning^50^. The titres of AAV were determined by QPCR analysis using specific primers targeting AAV genome (Supplementary Table 2) and the plasmid DNA standard curve. Eight-week-old male β-MDM2KO mice and its WT littermates were randomly assigned to intraperitoneal injection with AAV encoding DN-p53 or GFP control (1 × 10^12^ genomic copy number per mouse).

### Construction of the luciferase reporter vector and luciferase assay

A reporter plasmid encoding luciferase under the control of the mouse *PC* gene promoter was constructed by amplifying the promoter region spanning −92,280 to −91,186 bp (the distal promoter 2, the number of nucleotides are relative to the start codon) and −6,882 to −5,708 bp (promoter region consisting of the putative p53 RE) of *PC* using genomic DNA extracted from C57BL/6N mice as template and then subcloned into pGL3-Basic vector (Promega). The putative p53 RE within the promoter region were mutated by PCR-based site-directed mutagenesis using the WT vector as the template. All constructs were confirmed by DNA sequencing. The sequences of all the primers used for the vector construction are listed in Supplementary Table 2. MIN6 β-cells were co-transfected with the reporter vector and pRL-TK renilla luciferase vector (Promega) as transfection efficiency control using Lipofectamine 2000 (Life Technologies) for 6 h, followed by infection with adenovirus encoding p53 or GFP at 50 m.o.i. for 24 h. For glucose stimulation experiment, the cells were incubated with a KRB buffer containing 2.8 mM glucose for 1 h, followed by stimulation with glucose (20 mM) for 12 h. The cells were collected for measurement of luciferase activity using a dual luciferase reporter assay kit (Promega)^51^.

### Chromatin immunoprecipitation

MIN6 cells were infected with adenovirus encoding FLAG-tagged p53 or luciferase as control at m.o.i.=50 for 24 h, followed by crosslinking with 1% formaldehyde for 15 min at room temperature. The crosslinking reaction was stopped by the addition of glycine (final concentration=125 mM). The cells were lysed in a lysis buffer (20 mM Tris, 2 mM EDTA, 500 mM NaCl, 0.1% SDS, 1% NP40, pH 8.0) and were then sonicated to generate DNA fragments with average size of <1,000 bp. Size of the DNA fragment was checked by DNA gel electrophoresis. Total cell lysates were subjected to immunoprecipitation with agarose coupled with anti-FLAG antibody (Catalogue #A2220, Sigma, 15 μl agarose per 1 mg total cell lysate) overnight at 4 °C. The complex was washed three times with the lysis buffer and then eluted with elution buffer (1% SDS and 0.1 M NaHCO_3_). The crosslinks were reversed by incubating the complex at 65 °C overnight, followed by DNA purification using QIAquick PCR Purification Kit (Qiagen). The DNA fragments were analysed by PCR using specific *PC* or *MDM2* promoter primers (Supplementary Table 2).

### Morphological and immunohistochemical analysis

Pancreases were rapidly isolated from mice, fixed in 4% paraformaldehyde in 0.1 M phosphate buffer, and were then embedded in paraffin and cut into 5-μm-thick sections at 50-μm intervals. To determine the distribution of MDM2 in pancreas, pancreatic sections from β-MDM2KO mice and its WT controls were incubated with mouse anti-MDM2 and rabbit anti-VAMP2 antibodies, followed by staining with secondary FITC-anti-rabbit IgG- (Catalogue #65-6111, ThermoFisher, 1:500 for IHC) and Cy5-anti-mouse IgG-conjugated antibodies (Catalogue #A10524, ThermoFisher, 1:500 for IHC), respectively. To detect the expression of FLAG-tagged DN-p53 in pancreas, pancreatic sections from AAV-injected mice were incubated with mouse-anti-FLAG antibody, followed by DAB staining. To calculate β-cell mass, the ratio between the area of islet and the total area of pancreatic section was multiplied by the weight of pancreas. In all, 4 different parts of the pancreas and 3 tissue slides for each part (total 12 tissue slides per animal) were examined. For electron microscopic analysis, isolated islets were fixed in 2.5% glutaraldehyde in cacodylate buffer, osmicated in 1% osmium tetroxide, dehydrated and infiltrated, and polymerized in epoxy resin. Sections (100-nm thickness) were stained with 2% aqueous uranyl acetate and Reynold's Lead Citrate, and examined under a Philips EM208s transmission electron microscope. Two independent investigators blinded to sample identity analysed the pancreatic sections.

### Measurement of intracellular level of ATP

For measurement of intracellular ATP, isolated islets or MIN6 cells were incubated in the Krebs buffer with 2.8 mM glucose for 30 min, followed by stimulation with glucose (20 mM) for 10 min. The level of intracellular ATP was measured using the Luminescence ATP detection assay system (PerkinElmer) and normalized with protein concentration.

### Measurement of calcium influx

Isolated islets or MIN6 cells were loaded with Fura-2 calcium indicator (ThermoFisher Scientific) for 30 min, followed by stimulation with glucose (20 mM). The fluorescence ratios at 340/380 were measured using a calcium ion sensing system (IonOptix). The calcium levels were calculated from three islets of each mouse, and four mice were included in each group.

### Measurement of intracellular levels of metabolites

Levels of NADH, NADPH and OAA in the islets or MIN6 cells were measured with PicoProbe NADH Fluorometric Assay Kit (BioVision), PicoProbe NADPH Quantitation Fluorometric Assay Kit (BioVision) and Oxaloacetate Colorimetric/Fluorometric Assay Kit (BioVision), respectively, according to the manufacturer's instructions. The isolated islets with similar size were used for the above experiments.

### Measurement of OCR and ECAR

OCR and ECAR of isolated islets and MIN6 cells were measured in XF24 respirometer platform (Seahorse Bioscience XF24 Extracellular Flux Analyzer). A total of 80–100 islets per well or 1.2 × 10^4^ MIN6 β-cells were pre-incubated in KRB buffer containing 2.8 mM glucose and 1% FBS for 2 h at 37 °C without CO_2_. Glucose (20 mM) was used to stimulate cellular oxygen consumption, and oligomycin (5 μM) was used to inhibit ATP synthesis as indicated in each figure.

### Caspase 3 and MTT proliferation assays

Activity of caspase 3 was determined by Caspase-3 Fluorometric assay kit (BioVision, Catalogue #K105-200) according to the manufacturer's instructions. Briefly, MIN6 cells infected with adenovirus encoding luciferase or p53 for 24 h were solubilized in the lysis buffer. A unit of 50 μg total protein were incubated with DEVD-AFC substrate at 37 °C for 1 h, followed by measurement of fluorescent intensity (excitation=400 nm; emission=505 nm). For quantifying cell proliferation, the infected cells were incubated with 10 μl MTT (5 mg ml^−1^) at 37 °C for an hour. The cells were solubilized with DMSO, followed by measurement of optical density at 520 nm.

### Statistical analysis

Data are presented as mean±s.e.m. All experiments were repeated at least three times with representative data shown. Animal sample size for each study was chosen on the basis of literature documentation of similar well-characterized experiments, and no statistical method was used to predetermine sample size. Statistical significance was determined by unpaired two-sided Student's *t*-test or one-way analysis of variance with Bonferroni correction for multiple comparisons. A *P* value of <0.05 represents a significant difference in all statistical comparisons. Data analysis was performed using GraphPad Prism Software Version 5.0a (GraphPad, San Diego, CA).

### Data availability

The authors declare that the data supporting the findings of this study are available within the article and its Supplementary Information files.

## Additional information

**How to cite this article**: Li, X. *et al*. The MDM2–p53–pyruvate carboxylase signalling axis couples mitochondrial metabolism to glucose-stimulated insulin secretion in pancreatic β-cells. *Nat. Commun.* 7:11740 doi: 10.1038/ncomms11740 (2016).

## Supplementary Material

Supplementary Information Supplementary Figures 1-14 and Supplementary Tables 1-2
