# Supplementary material for: The MDM2–p53–pyruvate carboxylase signalling axis couples mitochondrial metabolism to glucose-stimulated insulin secretion in pancreatic β-cells
Source: Nat Commun. 2016 Jun 6;7:11740. doi: 10.1038/ncomms11740 (PMC4897763; doi:10.1038/ncomms11740)
Supplement: Supplementary Information — Supplementary Figures 1-14 and Supplementary Tables 1-2 [file ncomms11740-s1.pdf]

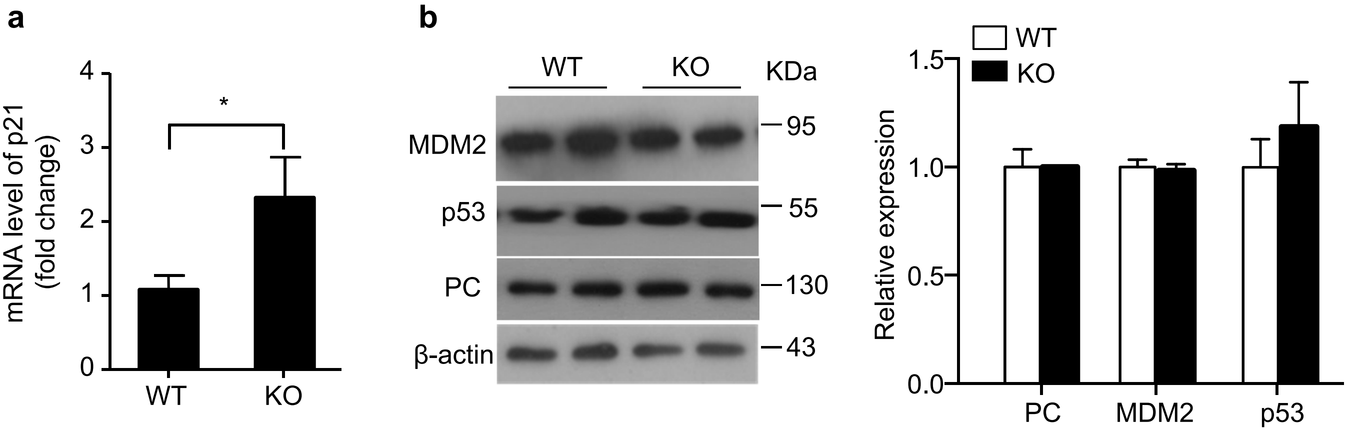

Supplementary Figure 1. Effect of MDM2 deficiency on expression of p53, PC and p21 in pancreatic islets and hypothalamus. (a) Quantification of mRNA level of *p21* in pancreatic islets isolated from 12-week-old male β-MDM2KO mice and its WT controls. Expression levels were normalized with β-actin and GAPDH (n=5-6). (b) Immunoblotting analysis of protein expression of MDM2, p53, PC and β-actin in hypothalamus of 6-week-old male β-MDM2KO mice and its WT controls. The right panel is densitometric analysis for the relative abundance of MDM2, PC and p53 normalized with β-actin (n=3). *$p<0.05$ (Student's t-test). All data are represented as the mean±s.e.m.

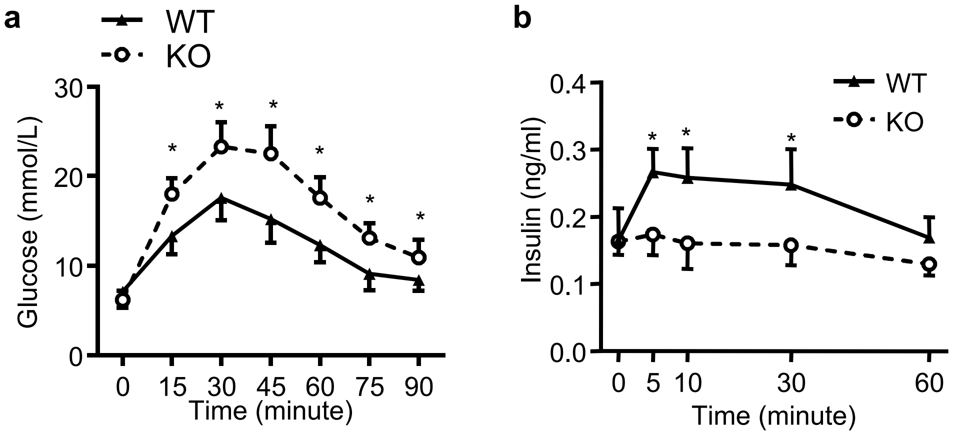

Supplementary Figure 2. Female β-MDM2KO mice exhibit glucose intolerance and defective GSIS. (a) GTT and (b) insulin secretion during the GTT (b) in 8-week-old female β-MDM2KO mice and its WT controls (n=4). *$p<0.05$ (Student's t-test). All data are represented as the mean±s.e.m. Note that RIP-Cre controls and non-RIP-Cre WT littermates exhibited similar glucose tolerance and GSIS.

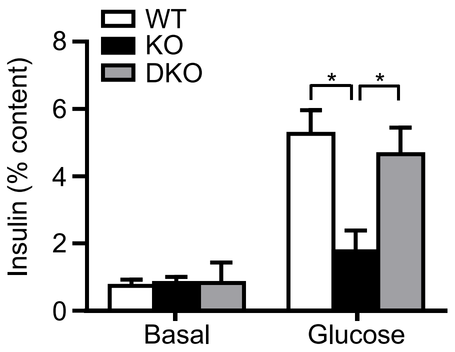

Supplementary Figure 3. Static GSIS in isolated islets of β-MDM2KO mice, DKO mice and WT controls. The isolated islets of 12-week-old male β-MDM2KO mice, DKO mice and WT controls were subjected to static GSIS as Figure 1g, followed by measurement of insulin concentration in the conditional medium and in the islets at 30 minute (n=5). *p<0.05 (Student's t-test). All data are represented as the mean±s.e.m.

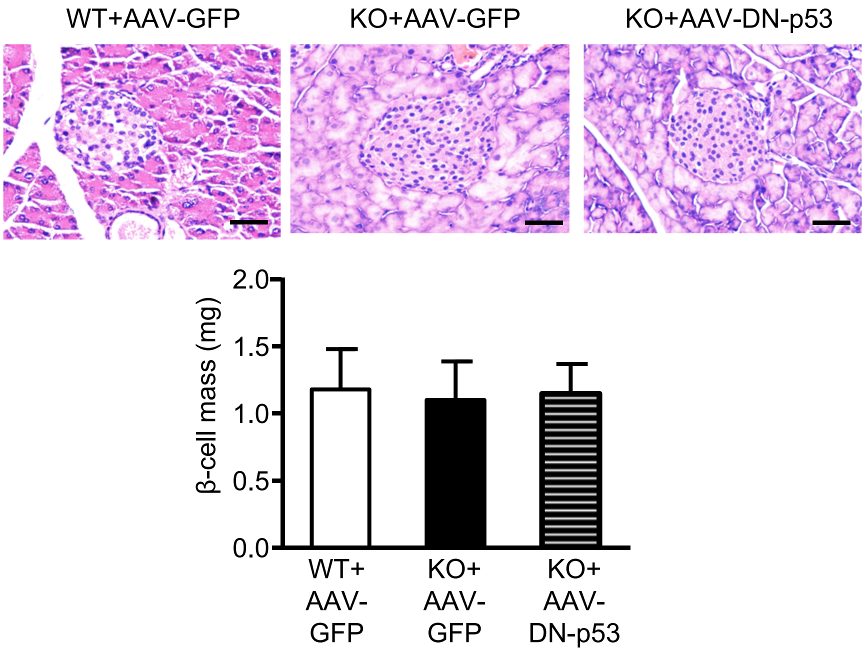

Supplementary Figure 4. Effect of AAV-mediated inactivation of p53 on β-cell mass. 8-week-old male β-MDM2KO mice and its WT controls infected with indicated AAV at a dosage of $1X10^{12}$ genomic copy for 3 week were used. Pancreatic sections from the above mice were subjected to H&E staining (magnification at ×400). The bar chart in the lower panel is the quantitative measurement of β-cell mass from the upper panel (n=6). Representative images were shown. Scale bar, 50 µm. All data are represented as the mean±s.e.m.

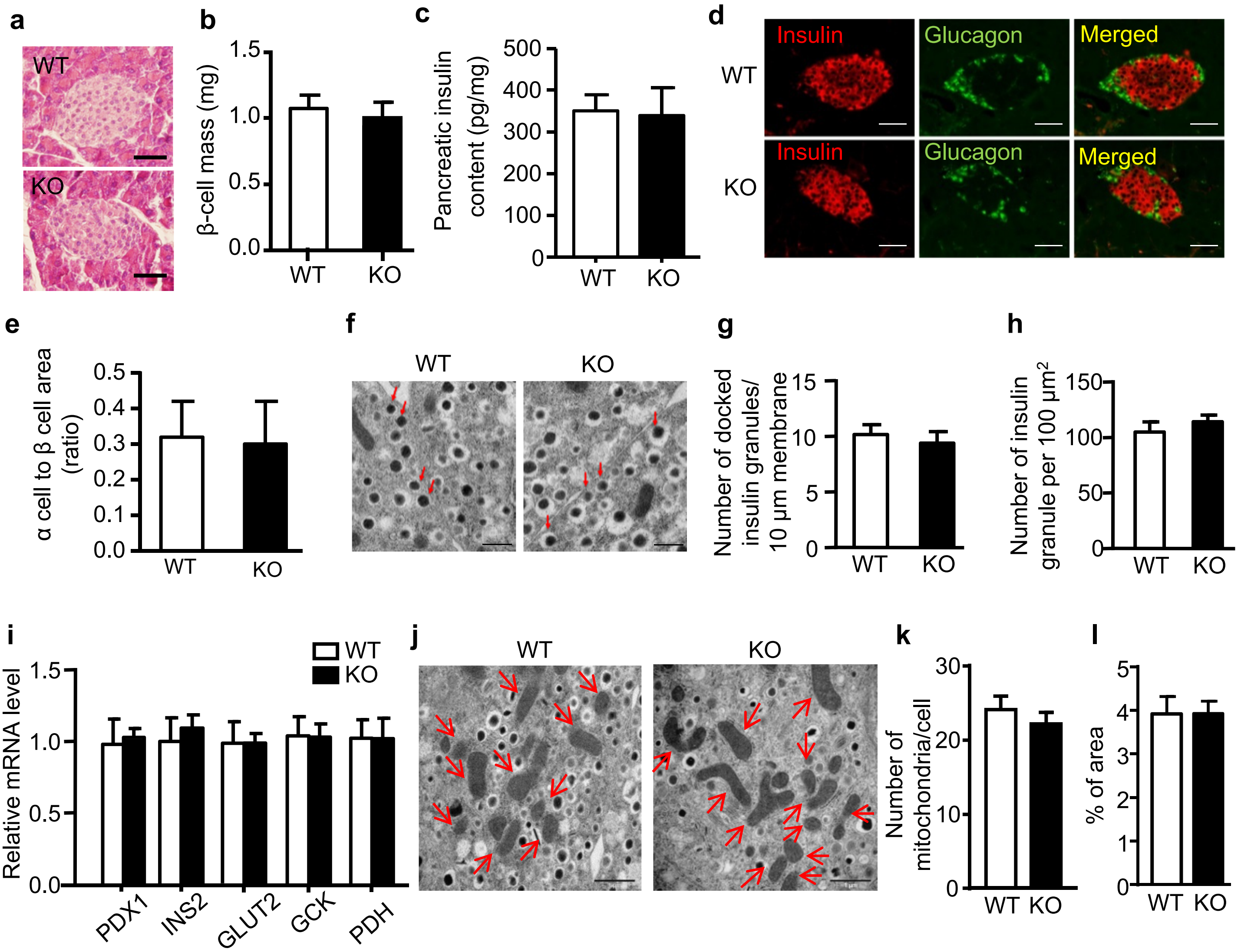

Supplementary Figure 5. Effect of MDM2 deficiency on insulin content, mitochondrial and β-cell morphology, insulin granules and gene expression profiles in β-cells. 12-week-old male β-MDM2KO mice and its WT controls were used. (a) H&E staining of pancreas with original magnification at ×400 (scale bar, 50 μm). (b) β-cell mass measurement from panel a. (n=8). (c) Pancreatic insulin content was measured by insulin ELISA. (n=6). (d) Immunofluorescence staining of insulin (red) and glucagon (green) of pancreas with original magnification at ×400 (scale bar, 50 μm). (e) The bar chart is the quantification of α-cell to β-cell ratio from panel d. (f) The isolated islets were subjected to electron microscopy analysis (scale bar, 1 μm). Docked insulin granules were denoted with arrows. Quantification of the number of docked insulin granules (g) and total insulin granules (h). (n=4). (i) Quantification for mRNA levels of *pancreatic and duodenal homeobox 1 (PDX1)*, *insulin II (INS2)*, *glucose transporter 2 (GLUT2)*, *glucose kinase (GCK)* and *pyruvate dehydrogenase (PDH)* in the isolated islets by real-time quantitative PCR analysis. All the target genes were normalized with *GAPDH* and *β-actin* and expressed as fold change relative to WT controls. (n=4). (j) Mitochondrial morphology in isolated islets examined by electron microscopy analysis (scale bar, 1 μm). (k-l) Quantification of the number (k) and percentage of area (l) of mitochondria. Mitochondria are denoted with arrows. (n=4). Representative images were shown. All data are represented as the mean±s.e.m.

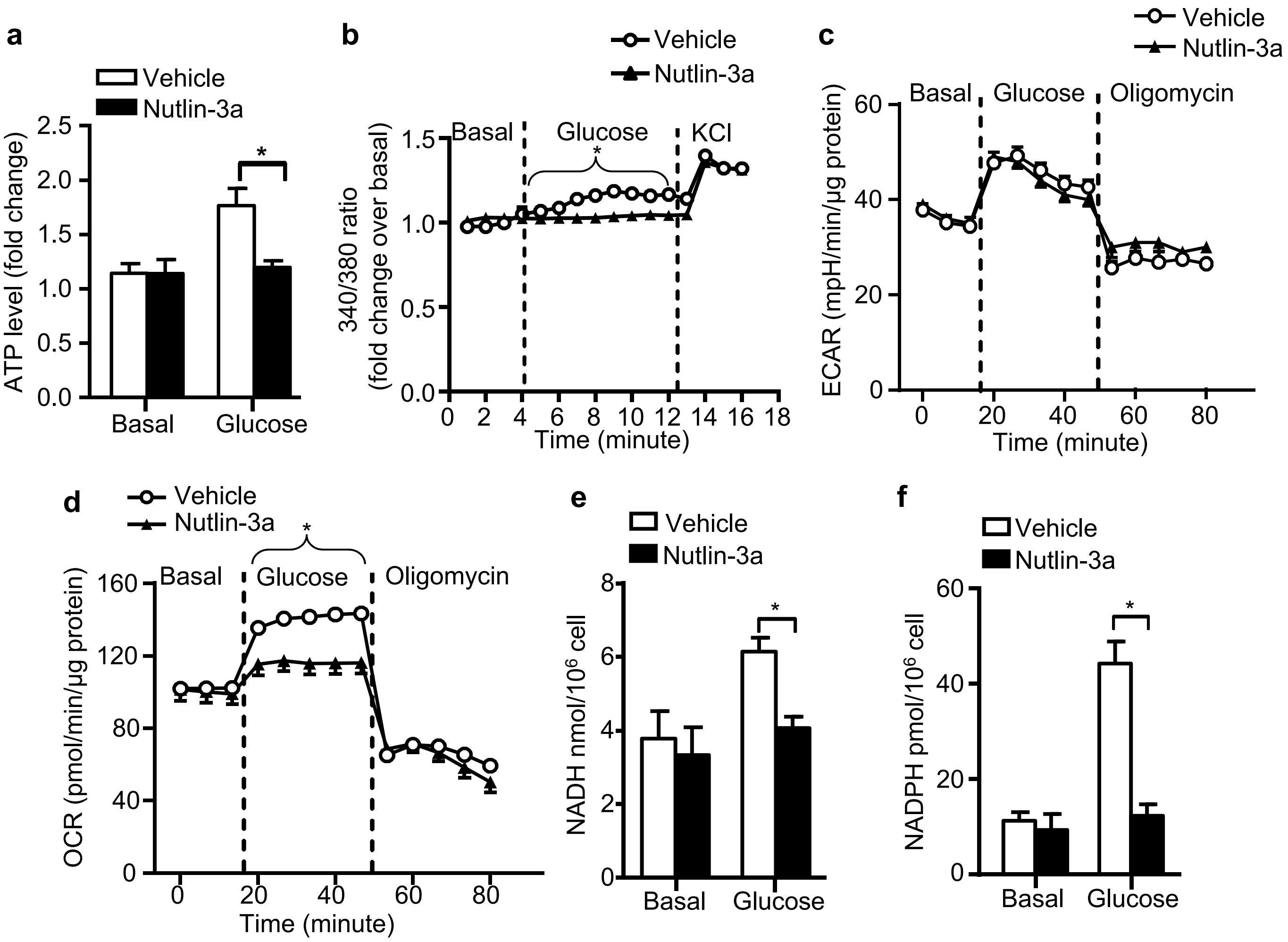

Supplementary Figure 6. Effect of nutlin-3a on mitochondrial metabolism in MIN6 β cells. MIN6 cells were pre-treated with nutlin-3a (10 µg/ml) or DMSO as vehicle for 6 hours. (a) Intracellular ATP levels upon glucose stimulation (20 mM) for 10 minutes. (b) Calcium influx in response to glucose (20 mM) and KCl (20 mM) stimulation. (c-d) ECAR (c) and OCR (d) in response to glucose (20 mM) and oligomycin (5 µM) stimulation. Intracellular level of NADH (e) or NADPH (f) in response to glucose (20 mM) stimulation for 30 minutes. *$p<0.05$ (n=4) (Student's t-test). All data are represented as the mean±s.e.m.

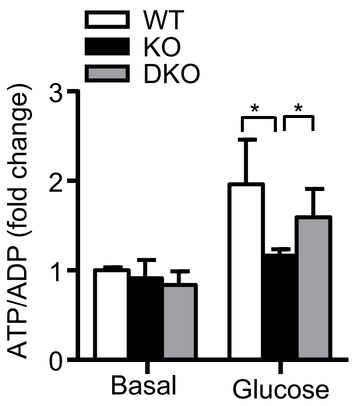

Supplementary Figure 7. Changes in ATP to ADP ratio in response to high glucose stimulation in islets of β-MDM2KO mice, DKO mice and their WT controls. Islets isolated from 10-week-old male β-MDM2KO mice, DKO mice and WT controls were used. Intracellular ATP/ADP ratio measured at 10 minute after stimulation with glucose (20 mM) using ADP/ATP Ratio Bioluminescence Assay Kit (Biovision, Catalog number K255-200). n=4. *$p<0.05$ (Student's t-test). All data are represented as the mean±s.e.m.

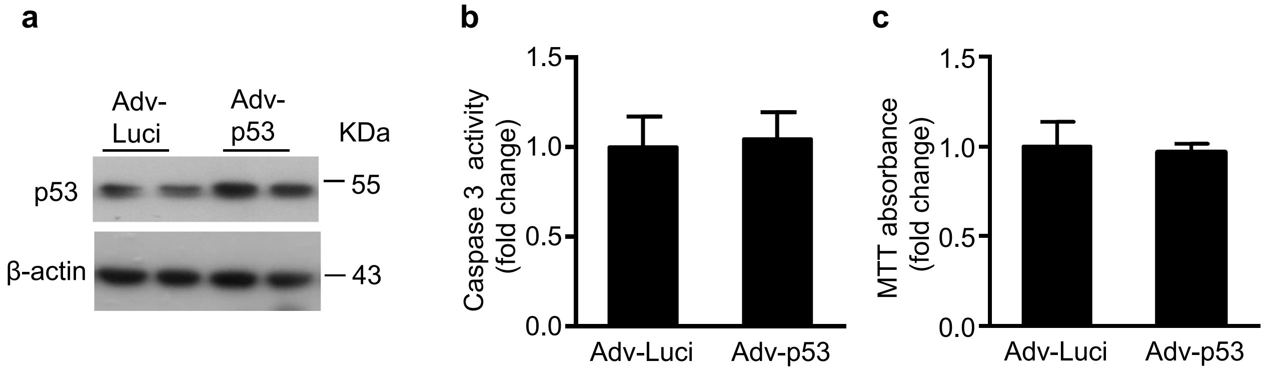

Supplementary Figure 8. Effect of p53 overexpression on apoptosis and proliferation in MIN6 cells. MIN6 cells were infected with adenovirus encoding luciferase (Adv-Luci) or p53 (Adv-p53) for 24 hours. (a) The infected cells were subjected to immunoblotting using an antibody against p53 or β-actin as indicated. (b) Activity of caspase 3 in the infected cells, normalized with total protein and expressed as fold change over the luciferase control. (c) Absorbance of MTT proliferation assay in the infected cells, and expressed in fold change over the luciferase control. (n=4). All data are represented as the mean±s.e.m.

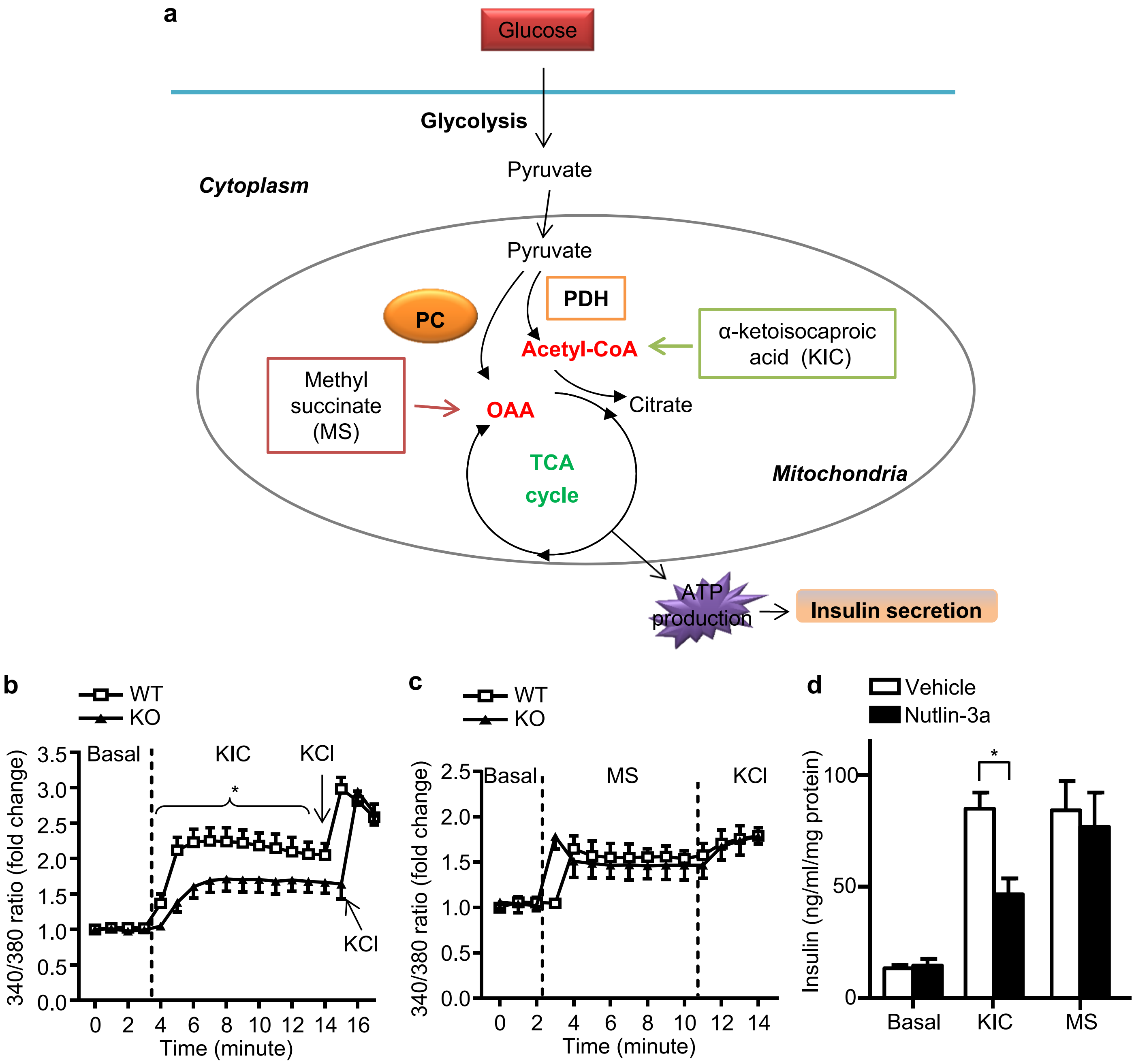

Supplementary Figure 9. Effect of MDM2 inactivation on the mitochondrial substrates-induced insulin secretion and calcium influx. (a) The schematic diagram illustrating how the mitochondrial substrates methyl succinate (MS) and α-ketoisocaproic acid (KIC) induce insulin secretion by generating OAA and acetyl-CoA in the TCA cycle, respectively. (b-c) Pancreatic islets were isolated from 10-week-old male β-MDM2KO mice and its WT littermates. (b) Calcium influx in response to KIC (10 mM, panel b) or MS (10 mM, panel c), and KCl (20 mM, panel b & c) in the isolated islets. (d) MIN6 β cells were pre-treated with nutlin-3a (10 μg/ml) or DMSO as vehicle control for 6 hours, followed by stimulation with KIC (10 mM) or MS (10 mM) for 30 minutes. Insulin levels in conditional medium were measured. *$P<0.05$ ($n=4$) (Student's t-test). All data are represented as the mean±s.e.m. Note that there was no difference in total insulin content among different treatments.

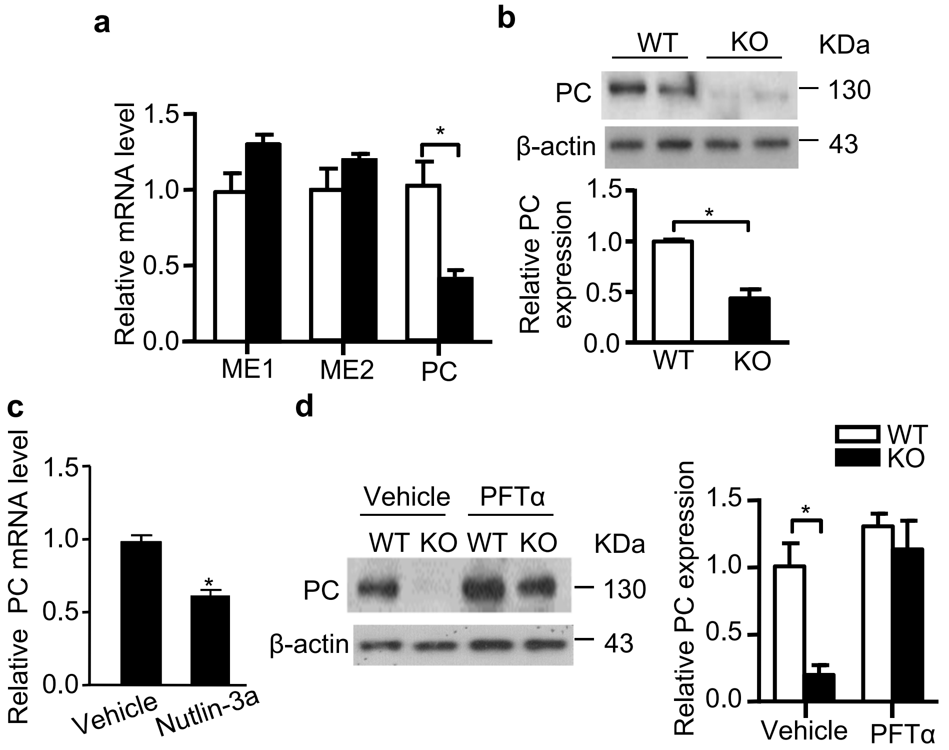

Supplementary Figure 10. The MDM2-p53 axis controls PC expression in β-cells. (a-b) Pancreatic islets isolated from 12-week-old male β-MDM2KO mice and its WT littermates were used. (a) Quantification of mRNA levels of *malic enzyme 1 and 2 (ME1 and ME2)*, *pyruvate carboxylase (PC)* by QPCR analysis. (b) Representative immunoblotting images for PC and β-actin from three independent experiment are shown. The bottom panel is the densitometric analysis for the relative abundance of PC normalized with β-actin. (c) QPCR analysis for mRNA levels of *PC* normalized with *GAPDH* and *β-actin* in MIN6 β cells treated with nutlin-3a (10 μg/ml) or DMSO as vehicle control for 24 hours. (d) Isolated islets from β-MDM2KO mice and WT littermates were treated with PFTα or DMSO as vehicle were subjected to immunoblotting using an antibody against PC or β-actin as indicated. Representative immunoblotting images for PC and β-actin from three independent experiment are shown. The right panel is densitometric analysis for the relative abundance of PC normalized with β-actin. *P<0.05 (n=4) (Student's t-test). All data are represented as the mean±s.e.m.

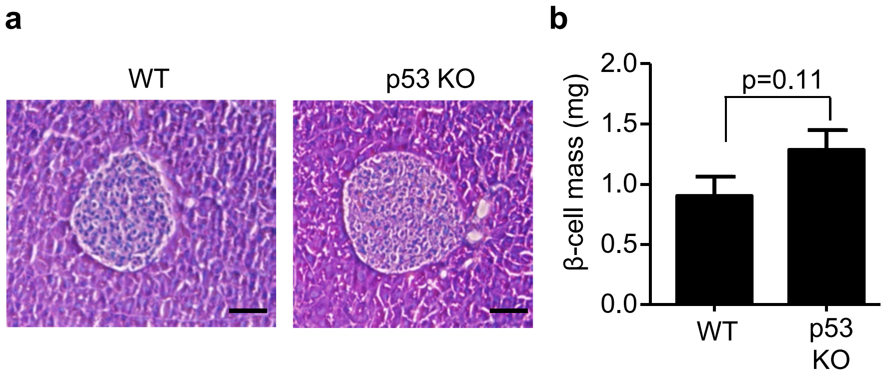

Supplementary Figure 11. Effect of β-cell deletion of p53 on β-cell mass in mice on normal chow. (a) H&E staining in the pancreatic sections of 14-week-old p53 KO mice and its WT controls on normal chow diet. Magnification at ×400 (scale bar, 50 μm). (b) Measurement of β-cell mass in panel a (n=6). Representative images were shown. The data are represented as the mean±s.e.m

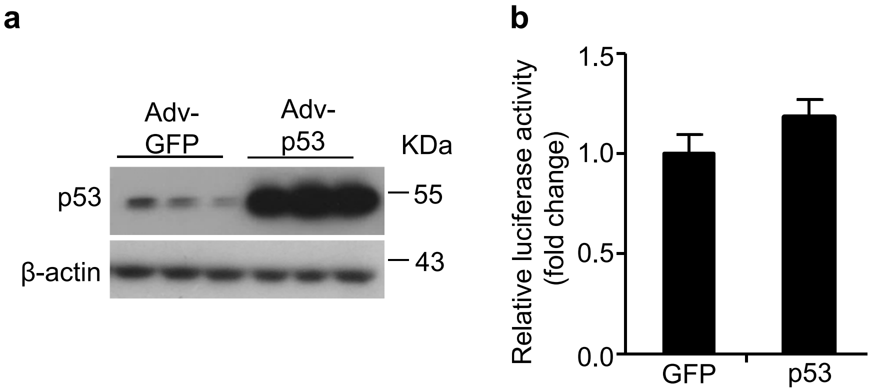

Supplementary Figure 12. Effect of overexpression of p53 on PC reporter luciferase activity in HEK293 cells. HEK293 cells were co-transfected with the wild-type PC firefly luciferase reporter and pRL-TK renilla luciferase reporter for 6 hours, followed by infection with adenovirus encoding GFP or p53 for 24 hours. (a) Immunoblotting analysis of p53 or β-actin in the cells. (n=6). (b) Relative dual luciferase activity in total cell lysate was measured, and expressed as fold change over GFP control. (n=5). All data are represented as the mean±s.e.m.

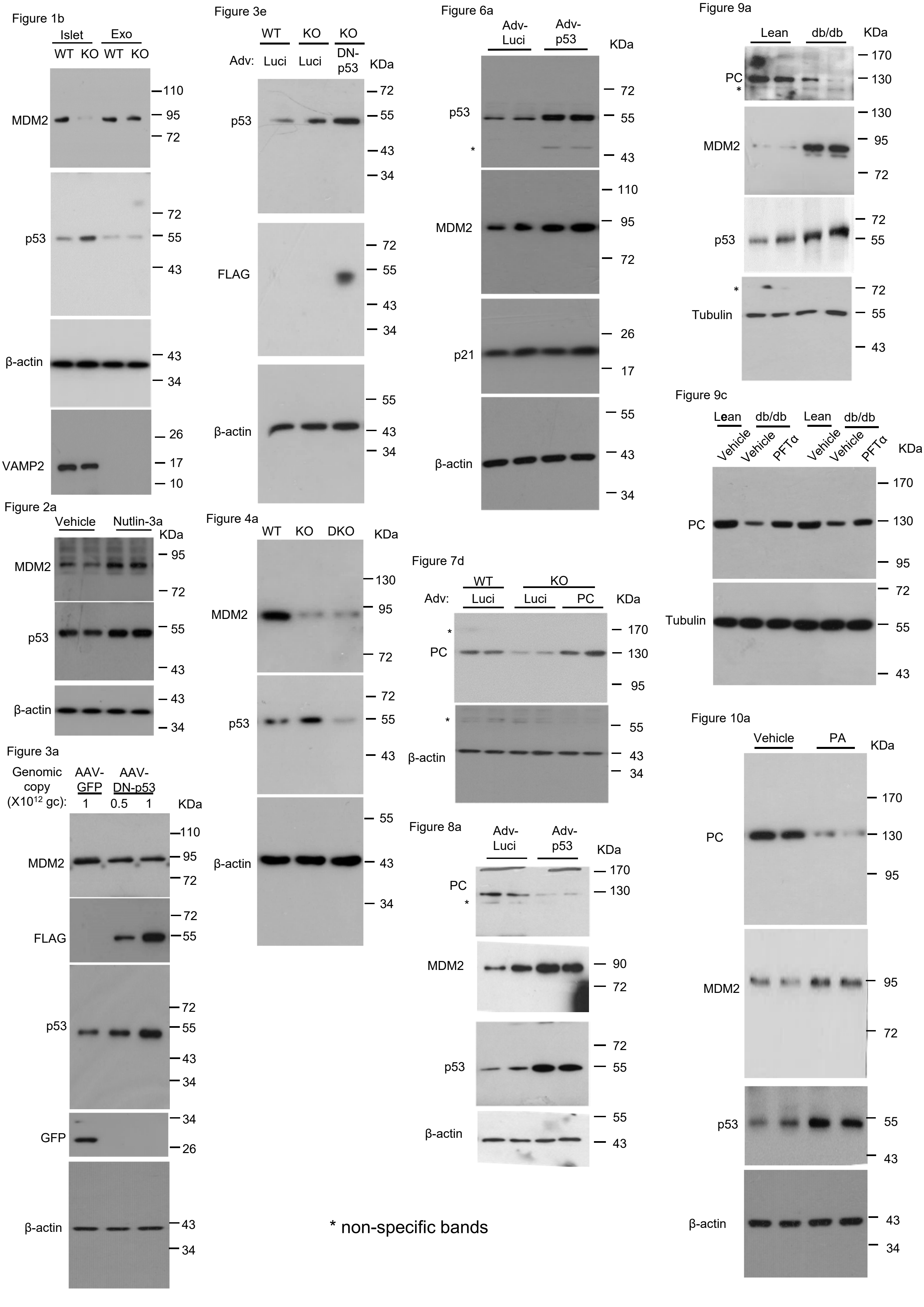

Supplementary Figure 13. Uncropped scans of the immunoblots (Figure 1-10)

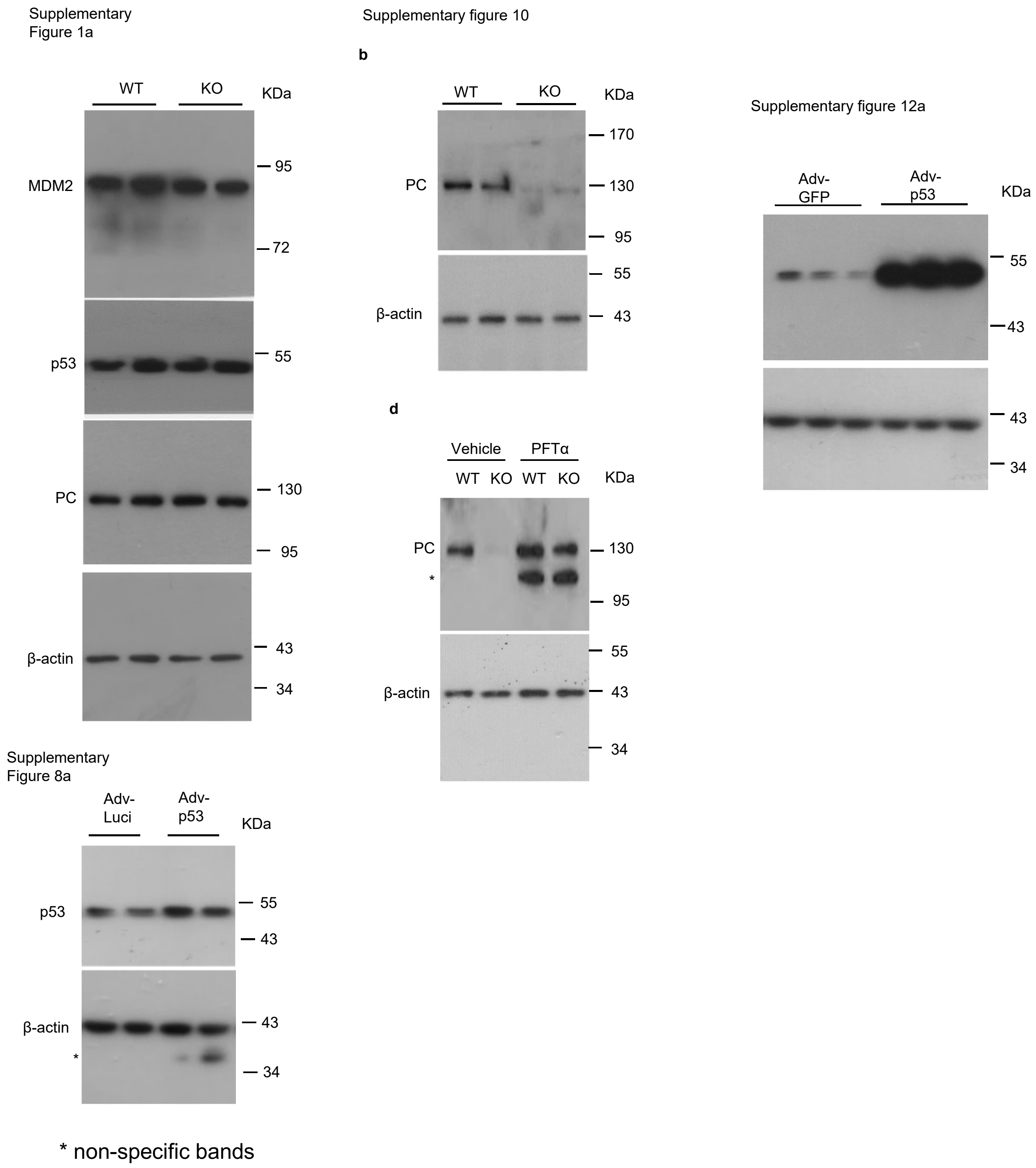

Supplementary Figure 14. Uncropped scans of the immunoblots (Supplementary Figure 1, 8, 10 and 12)

	6-week-old			20-week-old		
	WT	RIP-Cre	KO	WT	RIP-Cre	KO
Food intake (gram/day/mouse)	3.52±0.21	3.54±0.20	3.60±0.23	3.63±0.25	3.65±0.30	3.70±0.28
Body weight (gram)	20.10±0.10	20.09±0.12	19.29±0.27	25.21±0.29	24.96±0.21	24.97±0.33
Blood glucose (mmol)	5.0±0.8	5.2±0.9	5.6±1.0	5.7±0.9	5.6±1.0	6.3±1.2
Insulin (ng/ml)	0.41±0.12	0.39±0.14	0.35±0.14	0.52±0.14	0.48±0.15	0.44±0.21

Supplementary Table 1. Metabolic characteristics of β-MDM2KO mice and its WT littermates and RIP-Cre controls. 6-week-old and 20-week-old male mice were fasted for 6 hours before measurement of glucose and insulin levels. (n=5 for each group).

Gene	Forward (5'-3')	Reverse (5'-3')	Application
**PC**	GGGATGCCCACCAGTCACT	CATAGGGCGCAATCTTTTTGA	QPCR
**ME1**	AGTATCCATGACAAAGGGCAC	ATCCCATTACAGCCAAGGTC	QPCR
**ME2**	AGGAGAAGCTGCACTTGGAA	GCACCTGCCACTCCAATTAT	QPCR
**PDX1**	GGTATAGCCGGAGAGATGC	CTGGTCCGTATTGGAACG	QPCR
**INS2**	TGGAGGCTCTCTACCTGGTG	TCTACAATGCCACGCTTCTG	QPCR
**GLUT2**	CTTGGTTCATGGTTGCTGAAT	GCAATGTACTGGAAGCAGAGG	QPCR
**PDH**	GGGACGTCTGTTGAGAGAGC	TGTGTCCATGGTAGCGGTAA	QPCR
**GCK**	ATCTTCTGTTCCACGGAGAGG	GATGTTAAGGATCTGCCTTCG	QPCR
**MDM2**	GGGAGTGATCTGAAGGATCC	CTCATCTGTGTTCTCTTCTGTC	QPCR
**p53**	ACTGCATGGACGATCTGTTG	GTGACAGGGTCCTGTGCTG	QPCR
**p21**	GCAGATCCACAGCGATATCC	CAACTGCTCACTGTCCACGG	QPCR
**β-actin**	CGCCACCAGTTCGCCATGGA	TACAGCCCGGGGAGCATCGT	QPCR
**GAPDH**	CTCATGACCACAGTCCATGC	CACATTGGGGGTAGGAACAC	QPCR
**Human PC**	ATTGGTACCATGGATTACAAGGATGAC GACGATAAGCTGAAGTTCCGAACAGTC CAT	AATAAGCTTTCACCACAGTCTCCATCTTCAT GG	Cloning
**Mouse p53 (AAV)**	ATTGTCGACATGGATTACAAGGATGAC GACGATAAGACTGCCATGGAGGAGTCA CAG	AATAAGCTTTCAGTCTGAGTCAGGCCCCAC	Cloning
**Mouse p53 (Adv)**	ATTTCATGAATGGATTACAAG GATGACGACGATAAGACTGCCATGGAG GAGTCACAG	AATAGATCTTCAGTCTGAGTCAGGCCCCAC	Cloning
**DN-p53**	GACGGAGGTCGTGAGACACTGCCCCCA CCATGAGC	GCTCATGGTGGGGGCAGTGTCTCACGACC TCCGTC	Mutagenesis
**Mouse PC promoter p53 RE**	ATTCTCGAGGTAATTGTTCCAGAGCAG GTA	AATAAGCTTAGATAAGGAGTCTTCCTTGGA	Cloning
**Mouse PC promoter p53 RE Mut-1**	GGTTTTTACATGTGATGAAAGACGGCTC TGTGCTAGAGGACTAGTC	GACTAGTCCTCTAGCACAGAGCCGTCTTTC ATCACATGTAAAAACC	Mutagenesis
**Mouse PC promoter p53 RE Mut-2**	CATGCTCTGTGCTAGAGGACTAAAATG TCTGTCTGTCTGTCTGTCTC	GAGACAGACAGACAGACAGACATTTTAGTC CTCTAGCACAGAGCATG	Mutagenesis
**Mouse PC promoter p53 RE-ChIP**	GGCTGGTACAAGGTAATTG	CACATACACACAAAAAGAG	ChIP
**Mouse MDM2 promoter p53 RE-ChIP**	GGTGCCTGGTCCCGGACTCG CCGGG	CCGAGAGGGTCCCCCAGGGGTGTCC	ChIP

Supplementary Table 2. Primer sequences used for this study. Abbreviations: pancreatic and duodenal homeobox 1 (PDX1) and insulin 2 (INS2), glucose transporter 2 (GLUT2), glucose kinase (GCK) and pyruvate dehydrogenase kinase 1 (PDH) , malic enzyme 1 &2 (ME1&2), pyruvate carboxylase (PC), adeno-associated virus (AAV), adenovirus (Adv), response element (RE), dominant negative of p53 (DN-p53).
